# Integrating Flow Imaging Analysis and Single-Particle ICP-TOFMS for Comprehensive Micro- and Nanoplastic Characterization

**DOI:** 10.1021/acs.analchem.6c02328

**Published:** 2026-08-13

**Authors:** Sarah E. Szakas, Lyndsey Hendriks, Gunda Koellensperger, Benjamin T. Manard

**Affiliations:** † Chemical Sciences Division, 6146 Oak Ridge National Laboratory, Oak Ridge, Tennessee 37830, United States; ‡ University of Vienna, Faculty of Chemistry, Institute of Analytical Chemistry, Waehringerstrasse 38, 1090 Vienna, Austria

## Abstract

Flow imaging analysis (FIA), provides composition-agnostic morphological characterization. These measurements of particle size and shape are valuable to mass-based analysis, such as single particle inductively coupled plasma time-of-flight mass spectrometry (sp-ICP-TOFMS), which provides quantitative data on elements within particles. Using these two methods together enables informed use of geometric assumptions required by sp-ICP-TOFMS, as particle mass is typically converted to a particle diameter using assumed-spherical geometry. To validate this concept, parallel measurements to determine particle diameters were performed by FIA and sp-ICP-TOFMS on four particle suspensions: 300 nm polystyrene Eu-doped nanoparticles, 1 μm Fe-rich beads, 3 μm four element calibration polystyrene beads and 5 μm polystyrene beads. The Fe-particles obtained the highest percent difference from the manufacturer’s nominal diameter, as the mean diameter obtained by FIA was overestimated by 21% and sp-ICP-TOFMS underestimated the mean diameter by 20.7%. Two types of particles were selected to test the effect of varying the particle number concentrations (PNC) on sizing accuracy, and both methods accurately sized each particle population at the PNC expected. Single particle analysis of carbon has continued to be a popular research topic, with direct applications to environmental pollutants in terms of nano- and micro- plastics. Real-world plastic particles were studied, and FIA’s measured circularity values demonstrated that the particles deviated from spherical geometries, therefore sp-ICP-TOFMS data should be interpreted as mass-based rather than size-based. Combining these techniques enables improved interpretation of particle populations and evaluation of particle sizes.

## Graphical abstract

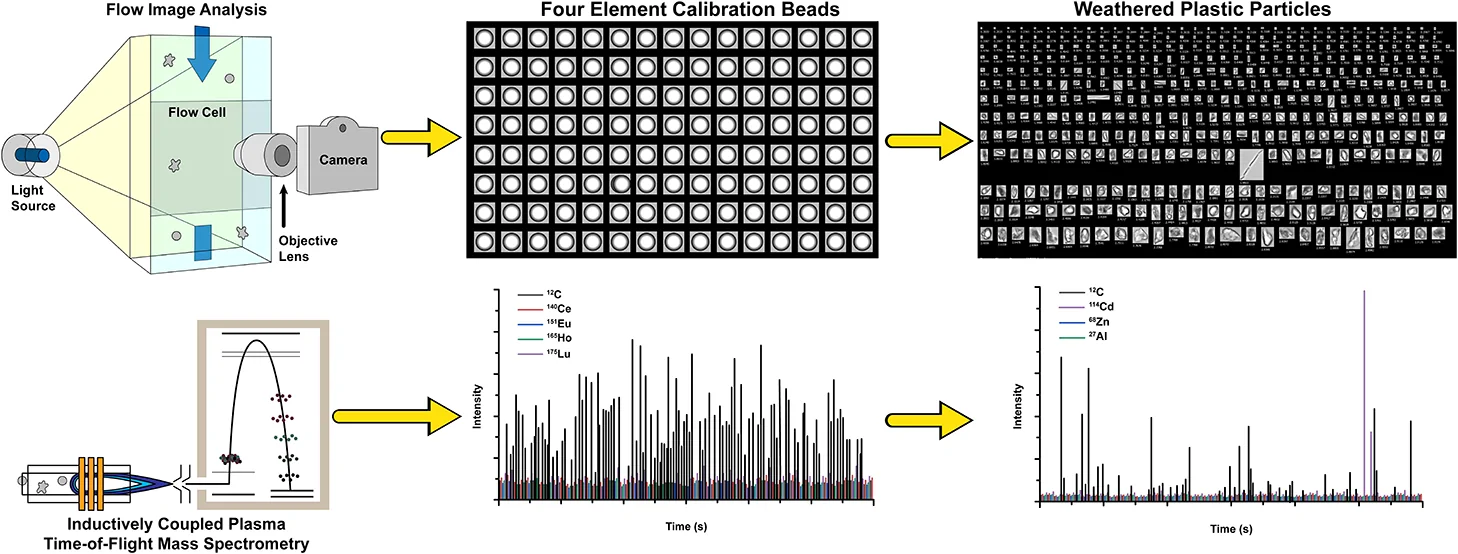

## Introduction

Micro- and nano- plastics are today recognized as ubiquitous environmental contaminants, with widespread and persistent presence within aquatic, terrestrial, and atmospheric systems.^1–5^ Microplastics are commonly defined as plastic particles smaller than 5 mm, while nanoplastics are generally considered particles with at least one dimension below 1 μm.^6^ These pollutants have raised significant concerns regarding their potential impacts on ecosystems and human health.^7,8^ Owing to their small size, and high surface-area-to-volume ratio, and diverse chemical compositions, these particles can interact with biological systems in complex ways, leading to potential toxicological effects.^9^ Understanding the physical and chemical characteristics of micro- and nano- plastics—particularly their size, shape, and composition—is therefore critical to assess their environmental fate, transport, and potential risks to ecosystems and human health.^10^

The importance of morphology in determining particle behavior and toxicity is well established. A well-known example is the case of asbestos, where fiber length (0.1–10 μm) and diameter (<0.5 μm) critically influence biological response: shorter, thicker fibers are associated with asbestosis, whereas longer, thinner fibers are linked to mesothelioma and lung cancer.^11^ In the context of micro- and nano- plastics, which breakdown into a variety of shapes and degradation states through their use and weathering, shape becomes particularly relevant.^12,13^ Increasing evidence suggests that micro- and nanoplastic shape and morphology determine toxicity, with several studies reporting that fibers, fragments, and spherical particles differ in their cellular uptake, persistence, and biological effects.^14–16^ In addition to morphology, particle size and particle number concentration (PNC) are equally important to assess.^17,18^

Nevertheless, the characterization of micro- and nano- plastics in complex matrices remains analytically challenging. To date, no single analytical approach can simultaneously resolve all relevant parameters including particle morphology, size, shape, number concentration, molecular, elemental, and isotopic composition. Among available analytical techniques, single-particle inductively coupled plasma mass spectrometry (sp-ICP-MS) has emerged as a powerful tool for quantifying elemental mass and number concentrations of microplastics on a single particle basis.^19–23^ In sp-ICP-MS, a highly diluted particle suspension (<10^5^ particles mL^–1^) is introduced and nebulized into the ICP, allowing individual particles to enter the plasma sequentially.^24^ Each particle is vaporized, atomized, and ionized, and a discrete cloud of ions is formed lasting a few hundred microseconds. These ion clouds are detected as transient signals that correspond to single particles, from which elemental mass, isotopic abundance, and particle number concentrations can be derived using appropriate calibration. As such, sp-ICP-MS enables discrimination between particulate and dissolved fractions (ionic backgrounds) and provides quantitative elemental data on each detected particle. Assuming a spherical shape, a known mass fraction from particle composition, and particle density (usually assumed to be equal to that of the bulk material), mass results can be converted to particle diameters.

This capability makes sp-ICP-MS particularly valuable for the study of micro- and nano- particles; however, it faces intrinsic limitations for plastic and polymer analysis. Detecting the main constituent of micro- and nanoplastics–carbon–is traditionally challenging by ICP-MS due to its high ionization energy and ubiquitous background. Although several groups have successfully demonstrated the utility of carbon-based sp-ICP-MS for proof-of-principle as well as applied studies, current limits of detection remain around 1 μm,^20–23^ which restricts the direct analysis of smaller particles such as nanoplastics. As a result, alternative strategies relying on the use of metal dopants,^25–28^ detection of polymerization catalyst residues,^29^ or intrinsic metal additives^30,31^ as proxies for detecting carbon have been explored.^32,33^ Furthermore, practical constraints related to transport efficiency, ionization yield, and the linear dynamic range of the instrument can complicate quantification across diverse particle sizes and compositions.^34^ Recent studies have also shown that the upper particle size detection for microplastics by sp-ICP-MS is currently limited to 20 μm.^35^

These challenges are especially critical when analyzing environmentally realistic samples, which are typically heterogeneous mixtures of different polymers, additives, and degradation products. Particles often exhibit irregular shapes or aggregation,^36^ and their composition can change through aging and weathering, further complicating accurate interpretation of sp-ICP-MS data. While particle diameters are an important metric, and are commonly referenced (e.g., PM2.5, PM10), interpretation of data using only an assumed spherical geometry may skew or hide true size distributions of particles that exhibit nonspherical geometries. Complementary techniques that provide shape and concentration information are therefore crucial to support and validate sp-ICP-MS results. Optical microscopy is useful for shape, color, and some surface features but is low-throughput, may not be able to distinguish transparent plastic particles, and often does not provide the resolution to see nanoplastics.^37^ Scanning and transmission electron microscopy techniques (SEM, TEM) provide detailed images of particle morphology but require extensive sample preparation and are also low throughput; usually only a small subset of particles within a sample can be analyzed. Dynamic light scattering (DLS) offers rapid, ensemble-based size data but assumes spherical geometry and struggles with polydisperse or irregular shape systems.

A more representative way to provide both particle morphology, number concentration, and elemental composition is to combine imaging techniques with ICP-MS. Previous attempts include microdroplet generator (MDG) setups, where a CCD camera is used to monitor droplet formation.^38,39^ In these systems, custom sample introduction setup allow the introduction of monodisperse microdroplets of approximately 50–90 μm in diameter, which are used as carriers for cells^40^ or nanoparticles^41,42^ or are doped with multielement solutions for quantification.^41–43^ In all cases mentioned, the primary goal was to measure droplet size and stability rather than to visualize the suspended analytes themselves. Nonetheless, Vonderach et al. presented a cell visible inside a droplet, marking the first demonstration that discrete objects within droplets could be optically observed prior to plasma introduction.^44^ This study laid the conceptual foundation for flow imaging analysis (FIA) in combination with sp-ICP-MS.

Several recent studies have combined sp-ICP-MS with orthogonal optical or spectroscopic techniques to image particles before they are delivered to the plasma, including Dynamic Image Analysis (DIA),^45^ flow cytometry (FC),^46^ and optical trapping coupled to Raman spectroscopy (OF2i-SP).^47^ These studies focus on proof-of-principle workflow, transport efficiency calibration, and evaluation of measurement uncertainty. Excellent agreement was demonstrated in size and established traceable particle number concentration measurements, but micrometer-sized well-characterized spherical microplastic standards were used exclusively. These studies did not address irregular morphologies, nanoplastics, or morphology-induced biases in mass and size estimation from sp-ICP-MS data.

The coupling of flow imaging analysis (FIA), also called flow imaging microscopy,^48^ to sp-ICP-MS builds on the concept of imaging flow cytometry, where cells are passed sequentially through a detection region for optical and/or fluorescence measurements. FIA is analogous to this; high-resolution images of particles or cells are sequentially captured as they pass through the flow cell. From these images, detailed morphological descriptors (e.g., diameter, area, circularity, aspect ratio, elongation) and particle number concentrations (PNC) can be extracted.^49^ No staining or labeling is required for FIA (although some platforms offer the ability to take white-light images and fluorescence measurements in tandem), and it allows direct, high-throughput analysis of particle suspensions. This capability enables direct visualization, morphological classification, and sizing. Moreover, it provides essential information to validate the spherical assumption underlying sp-ICP-MS-based size calculations.

Going beyond the validation of particle number concentration alone, visual information derived from particle imaging can be used to directly assess one of the central assumptions underlying sp-ICP-MS, namely the conversion of measured elemental mass into an equivalent particle diameter based on an assumed spherical (or other) geometry. Integrating FIA with sp-ICP-MS provides a comprehensive analytical strategy, combining morphological information with elemental and isotopic composition at the single-particle level. However, direct analysis of carbon-based particles by sp-ICP-MS remains challenging because of the high carbon background and low sensitivity. In contrast, FIA is unaffected by carbon detection limitations and enables direct visualization of both micro- and nano- plastics, irrespective of whether they are doped or undoped.

In this study, we present an analytical approach combining FIA and sp-ICP-TOFMS to improve the accuracy of micro- and nanoplastic characterization. A time-of-flight-based ICP-MS was employed to further extend analytical capabilities beyond conventional quadrupole-based ICP-MS, which is limited to sequential monitoring of one or two isotopes per particle. The simultaneous multielement detection enables full elemental fingerprinting of individual particles and supports applications requiring isotopic information, such as source attribution,^50^ nuclear forensics,^51^ or characterization of rare earth element-doped particles.^52,53^ Furthermore, the use of metal-based proxies enables the indirect detection of small nanoplastics. The workflow was first validated using well-defined spherical particle standards to determine the working range of both techniques as well as assess their accuracy and comparability. Building on these results, the method was then applied to a case study analyzing cryo-milled ocean-weathered plastic particles, representing environmentally relevant micro- and nano- plastics with irregular shapes and heterogeneous morphologies. Specifically, we evaluate (1) how FIA-derived shape descriptors can be used to verify the spherical assumptions typically applied in sp-ICP-TOFMS, (2) how particle number concentration recorded with both techniques can be compared, and finally, (3) the combination of both techniques to support accurate particle size and mass quantification.

## Experimental Section

### Particles Analyzed

#### Micro- and Nanoplastics and Standard Sample Descriptions

To explore the sizing capabilities and assess the working range of both approaches, dilute suspensions of spherical monodisperse particles were prepared from commercially available suspensions. The purchased particles spanned the size range from 300 nm to 5 μm and included: spherical europium-doped polystyrene nanoparticles (Bangs Laboratories, Fishers IN, USA, purchased via PolyScience Europe GmbH, Hirschberg an der Bergstrasse, Germany), Dynabeads MyOne Streptavidin T1 microparticles (Thermo Fisher Scientific Waltham, MA, USA), EQ Four Element Calibration Beads (Standard BioTools Inc., formerly Fluidigm, San Francisco, California, USA), and 5 μm polystyrene microparticles (Supelco, MilliporeSigma, Bellefonte, PA, USA). Table S1 presents an overview of these particles and their relevant characteristics, used to assess and compare the working range of the FIA and sp-ICP-TOFMS. These particle samples will be referred to throughout the manuscript as Eu-NP, Fe-MP, EQ, and PS-MP, respectively. All dilutions were prepared in ultrapure water (ASTM Type 1 18.2 MΩ·cm) after sonication, with 10 ng mL^–1^ Cs. Cs was used as an uptake standard in sp-ICP-TOFMS analysis to verify the suspension reached the ICP.

While composition, in terms of polymer type and trace elements used, were provided by the manufacturer, one important number necessary to compare the size of the Eu-doped particles with both methods was the amount of Eu doped per particle. In sp-ICP-TOFMS analysis, only the Eu is readily detected, and so the combination of the average mass of Eu detected per particle, along with the manufacturer reported size allowed us to estimate the percent Eu-dopant and therefore calculate an estimated size from TOF data. We calculated Eu to compose 1.68%, which is comparable to what has been calculated with other Eu-doped polystyrene particles previously reported in literature.^54^

#### Irregularly Shaped Environmentally Relevant Microplastics

In order to mimic environmentally relevant microplastics, ocean-weathered plastic debris characterized in a previous study were used as starting material.^55^ These samples were cryo-milled to produce irregularly shaped micro- and nano- plastic fragments, representative of environmental microplastics.^56^ Approximately five grams of each material were manually cut into small fragments prior to cryogenic milling. Cryo-milling was performed using a benchtop unit (Retsch GmbH, Haan, Germany) equipped with a 50 mL stainless-steel grinding jar and 3 mm × 25 mm stainless-steel grinding balls. Samples were precooled for 10 min, then milled at 25 Hz for two cycles of 2 min each, with an intermediate cooling period of 2 min between cycles. The produced powder was allowed to dry until further analysis. An unweighed portion of the cryomilled powder was transferred to a 50 mL Falcon tube, diluted in MQ, and spiked with 10 ng mL^–1^ Cs. The sample was vortexed for 30 s, then left for 5 min to allow big pieces to settle. Visible floating pieces were avoided when dilutions of this suspension were made.

#### Calibration Standards

Three different calibration series were prepared gravimetrically from single/multielement dissolved ionic standards (High Purity Standards, SC, USA) and carbon standard. An Au calibration curve, ranging from 0 to 100 ng g^–1^, was used to calculate transport efficiencies with the use of 100 nm Au particles (100 nm Ultra Uniform Gold Nanospheres, Nanocomposix, CA, USA). A multielement calibration curve was made, also ranging from 0 to 100 ng g^–1^, to quantify metals within individual particles. Finally, a calibration curve for carbon was made, using a Total Organic Carbon Standard (1000 ppm Total Organic Carbon from KHP, Inorganic Ventures, VA, USA) with concentrations ranging from 0 to 50 μg g^–1^.

### Instrumentation and Data Evaluation

#### Flow Imaging Analysis

FIA was performed using a FlowCam Nano (Yokogawa Fluid Imaging Technologies, USA), which combines optical microscopy with continuous flow, such that particles in suspension can be imaged in real time. Samples were introduced using a microsyringe pump equipped with a 250 μL syringe, operated at a flow rate of 20 μL min^–1^, and passed through an optical flow cell (60 μL) where images were rapidly acquired, at 110 frames sec^–1^, using a high-resolution monochrome CMOS camera. The system works as an oil-immersion flow imaging microscope with a 40× objective, utilizing a blue LED light (λ = 475 nm). The effective pixel size is 70 nm per pixel. The manufacturer states the size range as 300 nm up to 2 μm, and precision and accuracy are expected to decrease for measurement morphology and sizing nearing these limits. Before each day of analysis, reference particles with certified diameters of 0.717 ± 0.008 μm (Duke Standards NIST Traceable Polymer Microspheres, Thermo Scientific, California, USA) were run to set up the autofocus. Acceptable diameters for the reference particles ranged from 2 to 23% for all analysis herein, which resulted in sizing from 730 to 880 nm.

Particle images were processed using VisualSpreadsheet software (version 6.0.12.10, Yokogawa Fluid Imaging Technologies, USA). Images were automatically segmented based on grayscale intensity and edge detection. FIA directly measures particle size independently of elemental composition, enabling accurate sizing of particles in the approximate size range of 300 nm to 2 μm, according to the manufacturer’s specifications. The accuracy in sizing is also partly determined by the optical configuration, focus, and sample properties. There are a few options to calculate particle diameters, namely, equivalent spherical diameter (ESD), area-based diameter (ABD) and filled diameter (FD). Particle morphology can be characterized by descriptors such as aspect ratio, circularity, elongation, and perimeter-based metrics. In this work, ESD and circularity were evaluated. Particle number concentrations were calculated by normalizing the number of imaged particles to the analyzed sample volume. Samples were run until the total analysis time reached 10 min, or until 15,000 particle images were collected. Data was exported as comma-separated values (CSV) files containing particle-level metrics, with associated image files for visual verification and subsequent inspection. A simplified schematic of FIA using the FlowCam Nano is shown in Figure 1.

**Figure 1:**
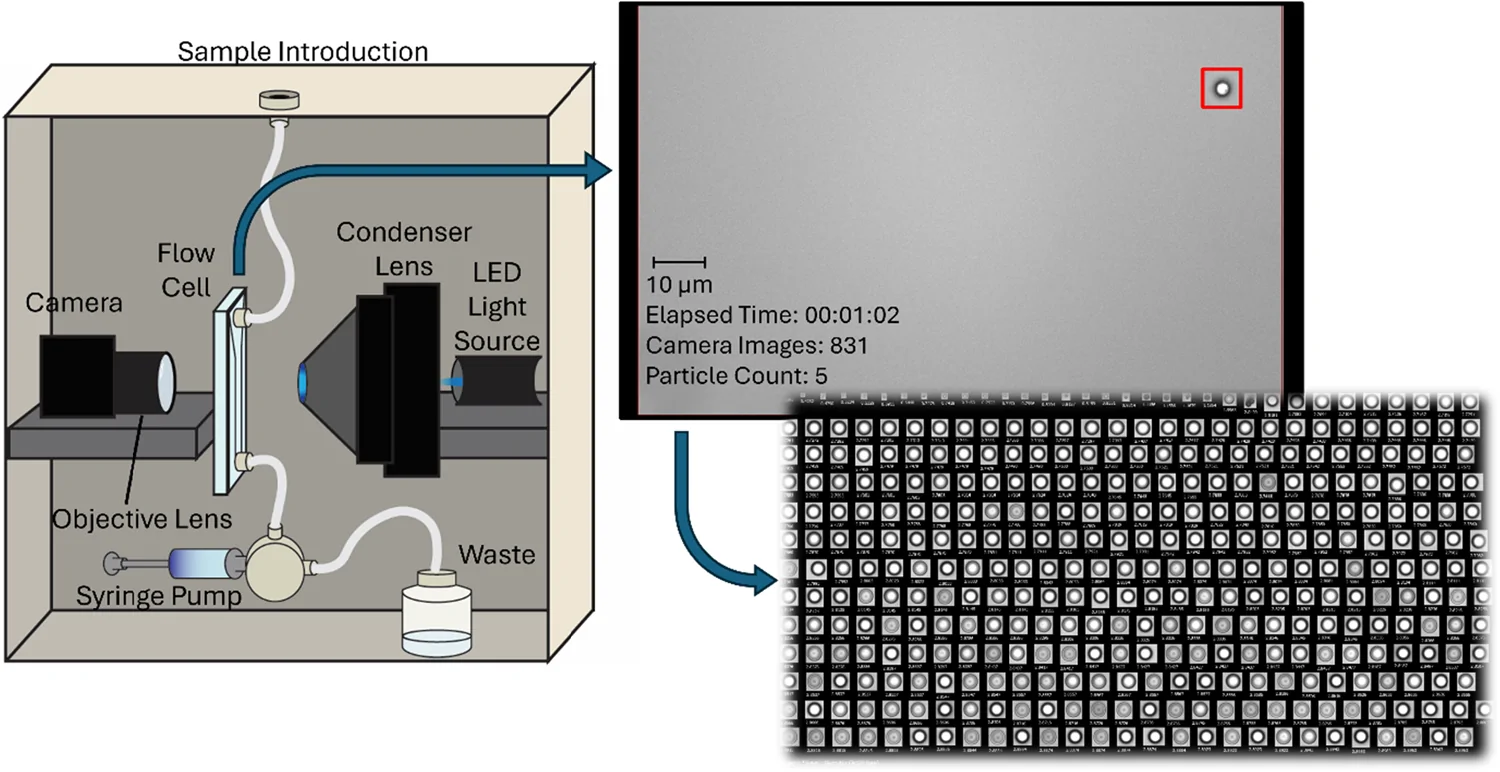
Flow image analysis (FIA) schematic showing the instrument (left). Following the first arrow, a zoom in on what the camera is imaging during analysis, from the flow cell. This middle image shows a “detected” particle, as shown by the red box around it. Finally, there is an example of the typical output from FIA, showing all particles imaged in a collage.

#### Single Particle ICP-TOFMS

Single-particle analysis was performed using an ICP-TOFMS (icpTOF R, TOFWERK, Switzerland) coupled to a high-throughput autosampler (microFAST SC, ESI, Omaha, USA) using the dedicated single cell CytoSpray spray chamber and CytoNeb200 (ID = 150 μm), which allows for improved transport efficiency of microsized particles.^57,58^ The ICP-TOFMS was tuned in “low-mass mode” to allow detection of carbon.^23^ Operating conditions for the ICP-TOFMS can be found in Table S2. Sp-data was recorded using the Single Particle module in TOFpilot version 2.15.14 (TOFWERK, Switzerland).^59^ Mass quantification was performed using ionic calibration standards and the transport efficiency–based approach described by Pace et al.^60^

Sp-ICP-TOFMS data was processed using TOF-SPI (TOF Single-Particle Investigator); a LabVIEW (National Instruments, TX, USA) program used for batch analysis of sp-ICP-TOFMS data.^61^ Data is treated using compound-Poisson thresholding^62^ to determine critical values (minimum signal needed to determine a particle from the dissolved background), calibration and transport efficiency, absolute sensitivity, and masses of user-selected elements within each particle event. TOF-SPI was also used here for split event correction, allowing for multiple data time bins to be summed into a singular particle event (necessary for large particles or particles with large signal spread).

Image analysis results and processed sp-results were exported as tabulated data files containing particle-level information. These outputs were used for subsequent statistical evaluation and comparison of both approaches. Further data processing and graphical representation was performed in OriginPro (OriginPro 2024 SR1, OriginLab Corporation, Northampton, MA, USA).

## Results and Discussion

### Analytical Validation Using Spherical Microplastic Standards

To establish and validate a robust analytical framework, the performances of FIA and sp-ICP-TOFMS were directly compared using commercially available spherical particle standards listed in Table S1. These well-defined particles are ideal for the direct comparison between the two techniques, as the spherical geometric assumption underlying mass-based particle size calculations for sp-ICP-TOFMS should be upheld. The insights gained from this step are crucial to be able to interpret results obtained for irregular, environmentally relevant microplastics, where particle morphology and heterogeneity play a more significant role. Examples of the data collected by FIA for each of the individual particle standards can be found in Figures S1–S4, while examples of the respective time traces obtained from sp-ICP-TOFMS are shown in Figure S5.

### Particle Size Determination

We first compare the particle diameters determined by both methods. Figure 2 shows the particle size distributions obtained by FIA (a) and sp-ICP-TOFMS (b) for the four spherical particle standards.

**Figure 2:**
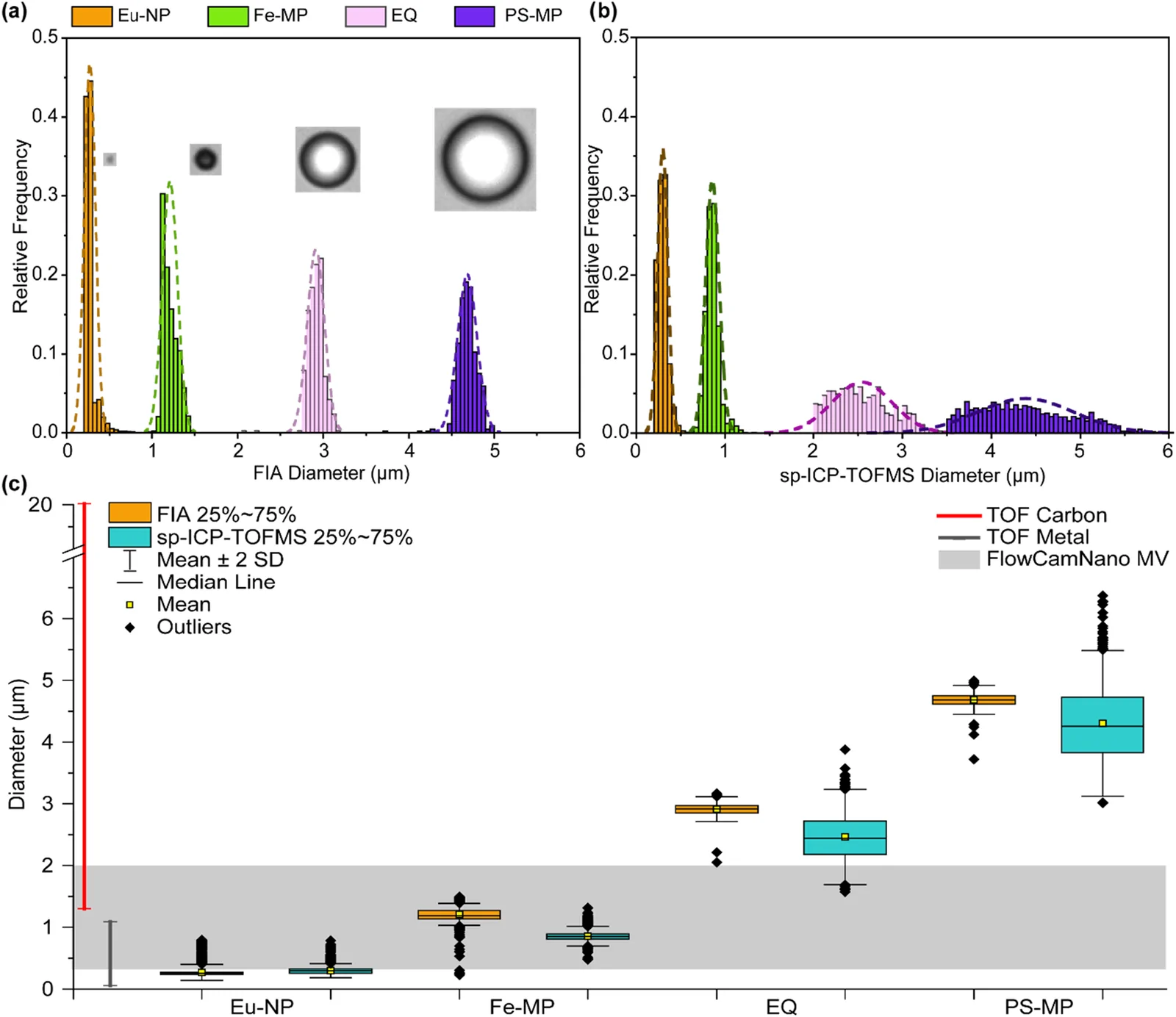
Size comparison of (a) FIA image-based diameters and (b) sp-ICP-TOFMS mass based calculated diameters for the Eu-NPs, Fe-MPs, EQ beads and PS-MPs. Included in (a) are select images obtained via FIA for each particle type measured and scaled the same. Both techniques show normal, monomodal distributions, which are indicated by the dashed lines. In (c) the techniques are compared to the diameters typically obtainable by both methods, indicated for sp-ICP-TOFMS by the left-most lines as TOF Carbon (red) and TOF Metal (gray), and indicated for FIA by the manufacturer specified size regime (shaded region).

To better determine the mean and median diameters of the particle populations chosen, thresholds were implemented to isolate each particle-type to avoid double events, particle agglomerates, degraded or aged particles, and potential sample carry-over from adversely affecting the determined sizes. The thresholds were determined with respect to the nominal values as well as the distribution of the particle diameters obtained, and were kept constant for both analysis types. For each particle type, a lower and upper diameter boundary were used; particles that were within these bounds, or thresholds, were used for analysis and plotted. Particles that fell outside these threshold values were, essentially, thrown away. Thresholds were set with respect to the distribution of particle diameters obtained. They are listed in Table 1 as the “Diameter Threshold Range”. After applying the thresholds, the means, medians, and number of particles either imaged by FIA or detected by sp-ICP-TOFMS were obtained and are reported in Table 1.

**Table 1:** Sample Statistics Obtained from FIA and sp-ICP-TOFMS

	FIA				sp-ICP-TOFMS			
	Eu-NP	Fe-MP	EQ	PS-MP	Eu-NP	Fe-MP	EQ	PS-MP
*N* (number of particles counted)	14,891	14,093	380	439	5064	3467	853	1346
Nominal Diameter (μm)	0.3	1	3.1	5.1	0.3	1	3.1	5.1
Median Diameter (μm)	0.25	1.2	2.9	4.7	0.30	0.85	2.4	4.3
Mean Diameter (μm)	0.27	1.2	2.9	4.7	0.30	0.86	2.5	4.4
Standard Deviation (μm)	0.07	0.09	0.1	0.1	0.06	0.08	0.4	0.6
Diameter Threshold Range	≤0.8	0.6–1.5	≥2.0	≥3.5	≤0.8	0.6–1.5	≥2.0	≥3.5
Percent of Particles within Threshold	99%	94%	95%	70%	100%	100%	99%	97%

To compare the diameters obtained between the two methods, we confirmed the particles used were spherical. Circularity was used as a shape descriptor, where a perfect circle yields a circularity value of 1, while increasingly irregular, elongated, or angular particles exhibit progressively lower values. Figure 3 plots the kernel density distributions for all four samples to visualize the overlap of particle measurements in terms of both circularity and diameter. Densities from each data set are normalized to their own maximum value, and the dark red spots indicate the highest overlap of data points. Over 92% of all particles measured, from every sample set, displayed circularity values ≥0.85, indicating that most particles were close to spherical and thus supporting the validity of the spherical assumption used for sp-ICP-TOFMS-based diameters.

**Figure 3:**
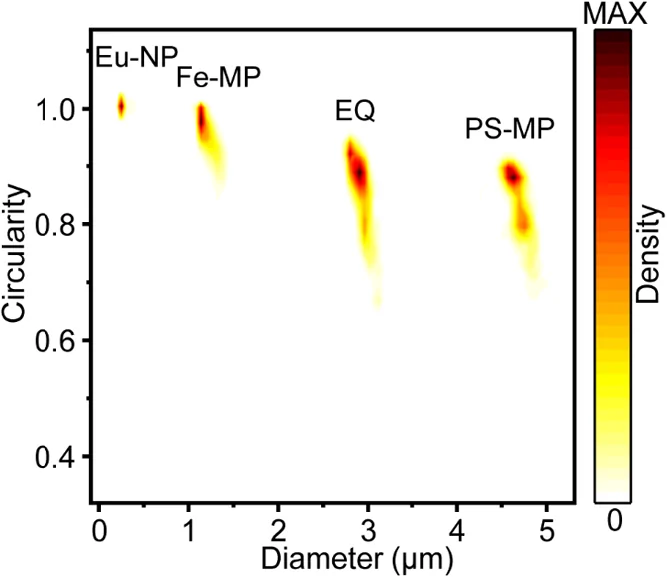
Circularity versus particle diameters for each sample, plotted as a kernel density estimates. The distributions show that most particles cluster within a narrow range of circularity (≥0.85) and diameter, indicating predominantly spherical particle morphologies.

Because many particles exhibited high circularity values, the assumption of spherical particle geometry was considered appropriate for subsequent diameter comparisons between the two analytical methods. Figure 2 shows that for all particle types, monomodal distributions are achieved, with broader distributions observed for sp-ICP-TOFMS compared to FIA measurements. Broader size distributions are commonly reported for sp-ICP-MS and arise from multiple sources of signal variability, including differences in droplet size during sample introduction, variations in particle position within the plasma that affect ion transmission, and counting statistics during ion detection.^63,64^ Similar behavior has been described in comparisons of sp-ICP-MS particle sizing with electron microscopy measurements. Overall, the mean diameters of all four particle types obtained by FIA matched the nominal (or previously published) values within ∼20%. The diameters obtained from each method are related to the manufacturer’s value using percent error; the percent error in the Eu-NP, Fe-MP, EQ, and PS-MP particles was 10.3%, 21.0%, 6.1%, and 7.7%, respectively. The larger polystyrene beads (EQ and PS-MP) had the most accurate results with respect to the manufacturer reported mean, despite both particles falling outside the reported working range (Figure 2c).

Diameters calculated for the EQ and PS-MP particles by sp-ICP-TOFMS had percent errors of 18.1% (EQ), and 13.6% (PS-MP) from the certificate diameter. While these diameters reported are systematically lower than expected, a few considerations regarding the measurements are worth highlighting. Determining EQ particles required detecting carbon, as well as the trace doped elements (Ce, Eu, Ho, and Lu), but both the EQ and PS-MP particles were sized solely on the carbon signal, with density (1.05 g mL^–1^) and mass fraction based off polystyrene (C_8_H_8_). While this should allow for accurate sizing, it should be noted that the doped amount of the REEs in the EQ beads is not known. While the observed undersizing of particles could, in principle, be attributed to inaccuracies in transport efficiency (TE), TE in this study was determined using a well-established and widely accepted procedure in sp-ICP-MS, namely the particle size method described by Pace et al.^60^ This approach accounts for differences in instrumental sensitivity by relating particle signals to dissolved ionic standards, rather than relying solely on particle number concentrations as in the particle frequency method. The particle sizing approach assumes that particles are completely vaporized and ionized in the plasma, and that equivalent instrumental sensitivity applies to both calibration particles and measured particles. This is a standard method for TE calculation and accounts for a range of particle sizes due to using the sensitivity factors from a known particle along with the dissolved ionic standards. However, it has been shown that the TE experienced by both dissolved standards and larger (micron-sized) particles are not directly translated to the TE calculated via the nanoparticles.^34,65,66^ In this study, 100 nm Au NPs were used for calibration while micron-sized carbon particles were analyzed, representing substantial differences in both particle composition and size, respectively mass and number of atoms. Carbon has a higher first ionization energy (11.26 eV) than gold (9.23 eV), which may result in lower ionization efficiency in the ICP. In addition, larger particles require more time and energy to fully vaporize and atomize compared to nanoparticles. Incomplete vaporization or reduced ionization efficiency may therefore lead to reduced signal generation per unit mass, which would result in an underestimation of particle mass and consequently particle diameter for the larger carbon particles.^34^ It has also been discussed that particles over 2.5 μm in diameter may not completely ionize, or ionize off-axis, resulting in incomplete digestion of the particle and a lesser signal – also leading to under-sizing.^19^ These EQ beads have also been sized in literature previously and have shown widespread distributions with diameters ranging from ∼2.9 to 3.4.^67,68^

It should also be noted that TE is typically determined using Au NPs due to their commercial availability, good characterization and high instrumental sensitivity under standard ICP-MS tuning conditions. In this study, however, the instrument was tuned for optimal detection of low-mass elements (i.e., carbon), which resulted in reduced sensitivity for higher-mass elements such as Au. This effect is evident in Figure S6, where diminished signal intensity and broadened distributions are observed for the Au NPs histogram used in TE determination. The reduced sensitivity for Au under these conditions may lead to an underestimation of the apparent particle signal, which in turn can bias the calculated TE. As TE is directly used in particle size calculations, such bias could contribute to the systematic undersizing observed for the analyzed particles. While this represents a compromise inherent to multielement optimization in sp-ICP-MS, the chosen tuning conditions were necessary to enable reliable detection of carbon-based particles. However, we see the most accurate sizing of the smallest particles (300 nm Eu-NPs), which are the closest in size to the Au NPs used. Finally, the lack of particle standard stability represents a broader and unresolved challenge in the field. Both EQ and PS-MP suspensions used here were opened for more than three years and exhibited signs of degradation, which likely contributed to altered particle size distributions and reduced particle counts. Given the cost and limited shelf life of certified particle standards, annual replacement is not always a solution, underscoring the need for improved standard longevity and guidance on storage stability.

Even though the instrument was tuned for carbon, the metallic or inorganic constituents (Eu and Fe) had greater sensitivity (see Table S3). Particle size detection limits for all carbon-based beads were determined by carbon’s critical mass of ∼1.1 pg C, which in turn resulted in a critical size of ∼1.2 μm. Any polystyrene-based particle needed to be at least ∼1.2 μm in diameter to be detected. This made it impossible to use carbon to measure the nanoplastic standards (Eu-NP) – as such, these particles were detected based on their Eu-signal. The Eu signal, with the mass fraction of 1.68% Eu, was then used (with the density of polystyrene) to determine the particle size by sp-ICP-TOFMS. The critical size for a particle with the same composition as the Eu-NPs, based on the mass of Eu, was ∼210 nm. This cutoff is seen in the distribution (orange distribution in Figure 2a) where the smallest particles are not detectable. The percent error in the mean of the determined diameters vs the nominal value was 0.7%, as the mean diameter was determined to be 298 nm. FIA analysis error was slightly higher, at 10.3%, which could be due to these particles being at the lowest end of the instrument specifications (gray shading in Figure 2c). Because these small particles are represented by a limited number of pixels, the accuracy of image-derived sizing and morphology measurements may be limited.

The largest error in determined diameter, for both FIA and sp-ICP-TOFMS, were the Fe-MPs, with percent errors from the nominal diameter of 21% and 20.7%, respectively. FIA analysis slightly oversized the Fe-MPs (mean of 1.21 μm) while sp-ICP-TOFMS slightly undersized the particles (0.86 μm). Like the Eu-NPs, the metal signal was used to size particles via sp-ICP-TOFMS, with an assumed Fe mass fraction of ∼26%, and density of 1.8 g mL^–1^. It should be noted that both of these numbers are assumptions, either from information by the manufacturer, or from previous studies utilizing the same type of beads. Therefore, the exact percent of iron oxide making up these beads is not inherently known and, if assumed to be higher than the actual weight percent, would lead to Fe-MP under-sizing. The critical size for Fe-MP particles was ∼457 nm. These particles have previously been reported to contain around 292 fg of Fe per particle.^69^ However, when measured here, they averaged 158 fg of Fe per particle–almost 2× less than expected. Possible explanations for this discrepancy include having less sensitivity for Fe due to using the minor isotope (^57^Fe was selected due to the large background on ^56^Fe in low-mass tuning) as well as the tuning method employed (to detect C). These particles also show discrepancies because they are not perfectly spherical, but rather look rough, as previously observed by SEM imaging,^69^ due to their streptavidin coating surrounding the Fe-core. Therefore, while the mass of Fe within the particles may not be accurate, its use to determine diameter is also limited due to particle morphology. FIA shows similar accuracy in sizing, with slight oversizing most likely due to the ‘rough’ outer texture, causing it to include more pixels within the particle image when approximating the diameter. This may be addressed through optimization of edge gradient detection, or using a different method of calculating diameter, but will be left for future experiments.

Despite fundamentally different measurement principles, general agreement was observed between FIA and sp-ICP-TOFMS-derived particle sizes for the spherical standards, confirming the complementarity of both approaches and the validity of the mass-to-size conversion under these conditions. To further evaluate the overall performance of FIA vs sp-ICPTOF under more complex conditions, all the particle stocks were combined into a single mixture and analyzed by both methods. Figure 4 shows the histograms of the diameters of all particles detected via FIA, and the diameters obtained by assumed-spherical particles via sp-ICP-TOFMS. The fitted curves from the individual runs are also overlaid on these plots. Every detected particle’s diameter from FIA analysis is plotted in Figure 4(a), which shows distinct distributions, with zero to minimal overlap. Overall, using the previous thresholding (with slight modifications to ensure no overlap, i.e., Fe-MPs were limited to 0.8–1.5 μm and EQ were limited to 2 to 3.5 μm), 99.2% of all particles would be classifiable by their diameter as one of the four particle types. To better showcase the benefit of using FIA and obtaining narrow diameter distributions, we also calculated how many particles fell within ± 2SD for each particle type (with respect to the normal curve obtained from the single particle standard measurements in Table 1). In total, from the 1089 particles sized, 94.4% of particles fell within ±2SD for each particle type. Using only particles that fell within the ±2SD for each particle type, it was estimated that the composition of the mixture was 6.7% Eu-NP, 41.1% Fe-MP, 25.6% EQ, and 20.9% PS-MP. This shows the wide range of sizes accessible with FIA analysis, however, does illuminate that perhaps, when smaller particles or particles nearing the detection limits of the camera are in mixtures, they may not be recorded as shown by the underrepresentation of the Eu-NPs, for example.

**Figure 4:**
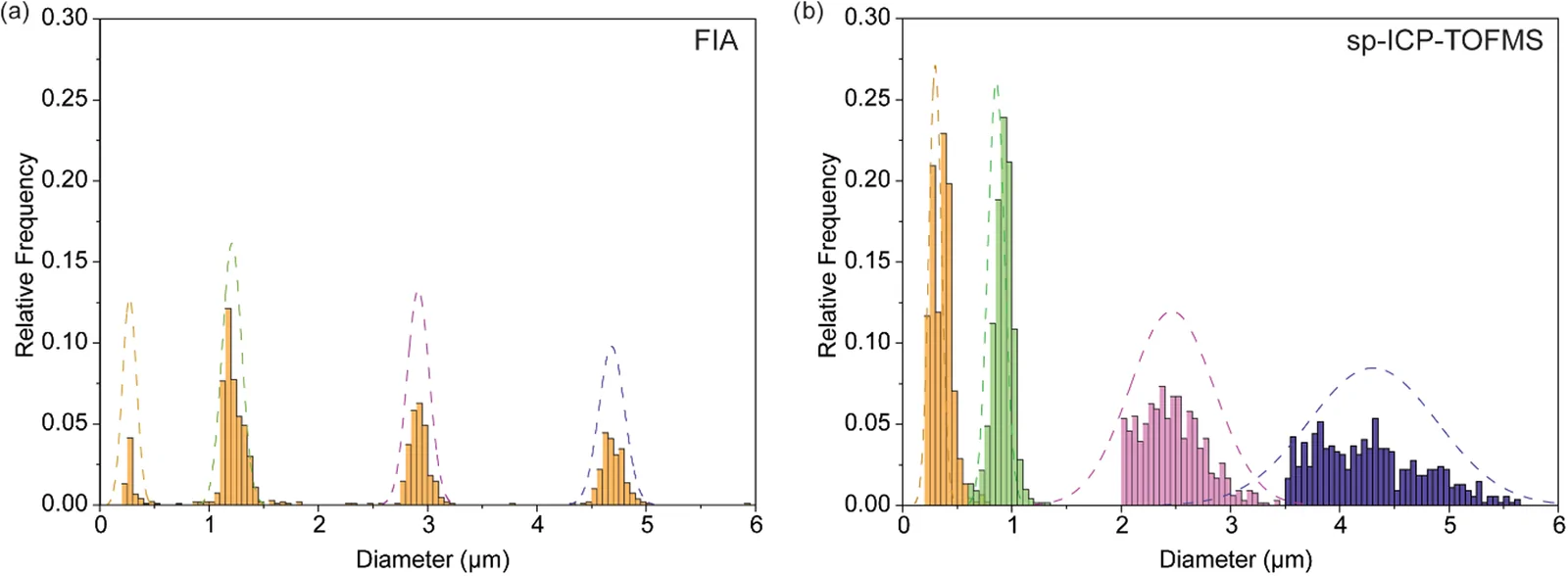
Histograms of mixtures containing all four particle standards for both (a) FIA and (b) sp-ICP-TOFMS. All four particles show distinct populations for both methods, with the curves obtained from the individual mixtures overlaid to show accuracy in sizing. Because particles are only told apart by their diameter in this FIA analysis, all diameters are plotted in orange in (a). Since sp-ICP-TOFMS sizes were element dependent, Eu-NP (orange), Fe-MPs (green), EQ (pink), and PS-MPs (purple) are all identified. Both graphs are plotted as relative frequency, where there is good agreement between both methods on the overall composition of the mixture in terms of number of each particle type.

Thresholds were also used for sp-ICP-TOFMS analysis of the particle mixture; however, the thresholds were applied to individual particle types rather than to every particle detected (was done for the FIA particle mixture). Figure 4(b) therefore shows only sp-ICP-TOFMS diameters after thresholding. For Eu-NPs and Fe-MPs, the thresholds described in Table 1 were implemented after sizing every particle containing only Eu or Fe. Even with using the thresholds, 100% of the “single-metal” Eu-NP and 96% of the Fe-MP particles were plotted (1205 Fe-MPs and 454 Eu-NPs). EQ particles were detected via C, Ce, Eu, Ho, and Lu and particles consisting of solely C were designated as PS-MPs. After it was determined that there were 655 EQ and 544 PS-MPs. Single particle analysis revealed wider distributions of particle sizes determined by carbon content—as seen in the individual runs. These distributions match the expected spread from the individual samples, but the threshold implementation does show a severe cutoff for the EQ distribution. The number of particles present within ±2SD was used to compare the composition of the mixture to FIA. As an aside, for the EQ and PS-MP particles, the lower threshold value (2 and 3.5 μm, respectively) were used as they were more constrictive than the calculated −2SD value. With a total of 2906 particles after thresholding, the composition of the mixture was determined to be 12.8%, 34.6%, 21.7%, and 18.4% in order of smallest to largest particle type. The smaller Eu-NPs were clearly more accurately determined via sp-ICP-TOFMS detection, but there is good agreement between the two methods regarding the Fe-MP, EQ, and PS-MP beads.

### Particle Number Concentration

Accurate PNC determination requires careful optimization of acquisition parameters for both techniques, as both rely on the assumption that individual particles are detected as discrete events during the measurement run. In the FIA approach, PNC depends on the relationship between flow rate, field of view (FOV), and image acquisition frequency. If the image acquisition rate is too high relative to the flow rate, particles may remain within the camera’s FOV across consecutive frames and will be recorded multiple times, resulting in oversampling and artificially elevated PNCs. On the other hand, if the acquisition rate is too low, portions of the sample volume may pass through the FOV without being imaged, leading to particle undercounting. In addition, suboptimal capture and segmentation settings can lead to further bias: closely spaced particles may be identified as a single object, underestimating PNC, whereas single particles may be wrongly segmented into multiple objects, overestimating PNC. Processing settings such as the distance to nearest neighbor (DNN) is particularly critical, as it defines the minimum separation required for two particles to be counted independently. Slight underestimation can also be explained due to particles passing through the camera that remain out of focus and are therefore not imaged (or counted). Similar considerations apply to sp-ICP-TOFMS: if particle arrival rates are too high relative to the acquisition time resolution, signal overlap can occur, leading to particle undercounting and artificially low PNCs. This is typically addressed through sample dilution, adjustment of sample uptake rates, or increased acquisition speed, ensuring that individual particle events remain temporally resolved.

To compare PNCs of particle suspension from FIA to sp-ICP-TOFMS, three different mixtures containing the 3 μm EQ four element calibration polystyrene beads (EQ) and the 5 μm polystyrene particles (PS-MP particles) were prepared. These two particle types were chosen for two main reasons. The first is that for both methods, EQ beads are robustly detected and sized. The second is that we wanted to use a particle standard that used the same sp-ICP-TOFMS sizing technique (size based on the carbon signal). It is important to note that PNCs of the stocks were not fully characterized via any method before these analyses. The exact PNCs of the polystyrene-based EQ and PSB particle stocks are expected to be different than stated due to their degradation, as seen from the individual runs. Furthermore, using ‘expected’ PNCs based on weight percent (PS-MPs) are likely to show greater inaccuracy than the particle types with a manufacturer-listed PNC (Eu-NPs, Fe-MPs, and EQ), shown in Table S1. In all three suspensions, the amount of EQ was kept at a constant PNC, while the PS-MP particles were added at three different volumes.

Thresholds were again used for both types of analysis; in short, in FIA, particle with diameters between 2 and 3.5 μm were considered EQ, and particles with diameters over 3.5 were considered PS-MP. In sp-ICP-TOFMS analysis, EQ beads were determined with codetection of C with Ce, Eu, Ho, and Lu, and if they were above 2 μm. An example time trace from Mixture 1 is shown in Figure S7. PS-MPs contained solely C and had to be above 3.5 μm. The number of particles detected that were classified as EQ and PS-MP are shown in Table 2. The ratios of EQ:PSB were compared to ascertain any bias on the results of the mixtures from either technique, as shown in Table 2. Figure 5 shows the histograms of each of the mixtures, after thresholding, for both FIA (left) and sp-ICP-TOFMS (right). Again, because no particle compositional information comes from FIA, essentially all particles with diameters greater than 2 μm are plotted. While the thresholds allow us to determine mean diameters for both particle populations, there is no way to separate out which particle belongs to which if overlap occurs. Visually, the mixtures look to be in good agreement. The overall mean diameter for both methods for the EQ beads in Figure 5(a) was 2.92 ± 0.09 and 4.7 ± 0.1 for the PS-MPs, while in Figure 5(b) it was 2.5 ± 0.4, and 4.3 ± 0.5, respectively.

**Figure 5:**
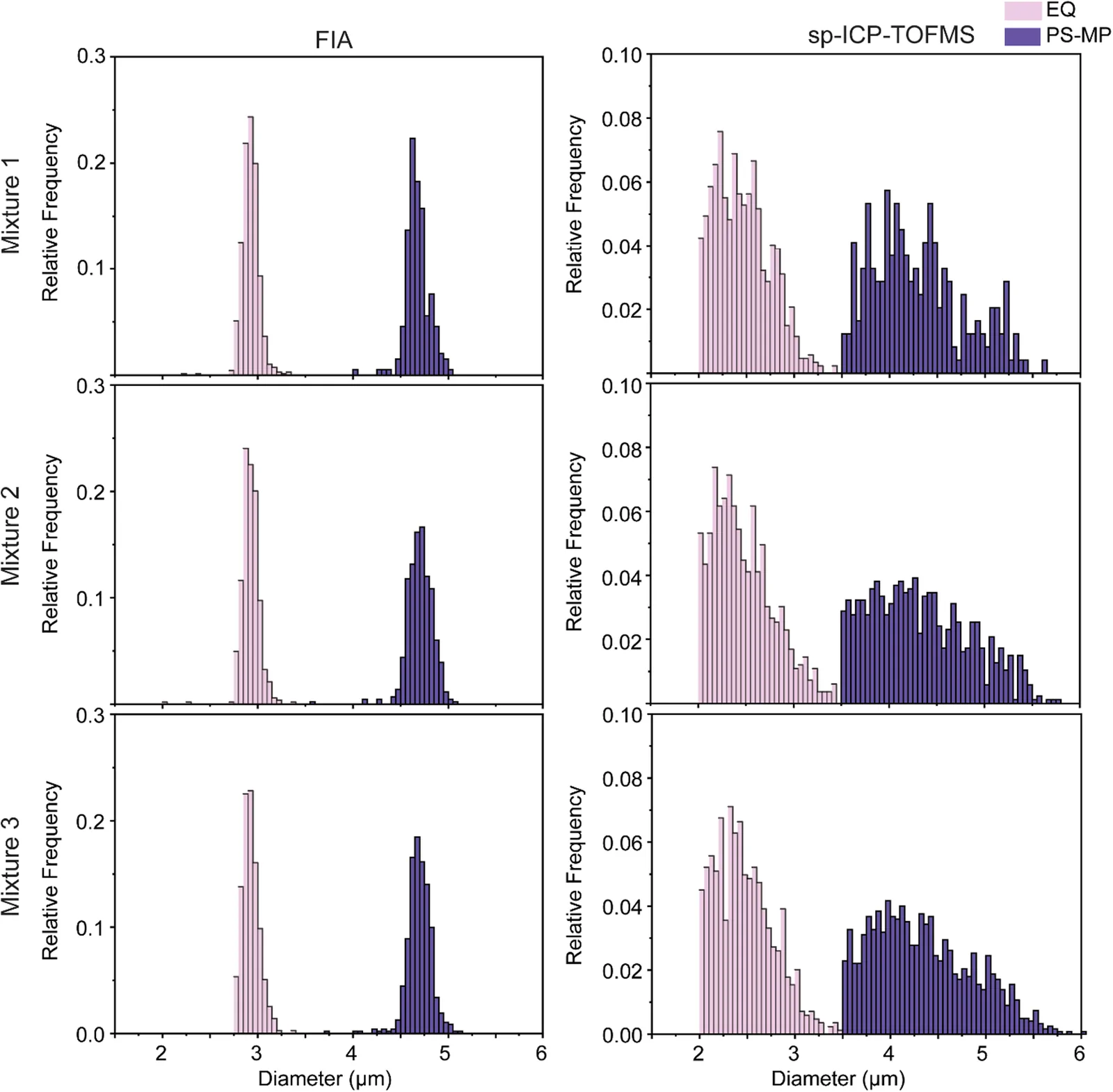
FIA (left) and sp-ICP-TOFMS (right) results for Mixtures 1, 2, and 3, where the PNC of EQ beads remain constant and the number of PS-MP particles increases. Both analyses show the same fraction of each of the particle types in all three mixtures, even when distributions obtained by sp-ICP-TOFMS are wider than those obtained by FIA.

**Table 2:** Mixtures of EQ and PS-MP Particles Showing the Count (Number of Particles That Fit Thresholding for Each Particle Type), PNC, and the Ratio of EQ:PS-MP

	FIA					sp-ICP-TOFMS				
	EQ		PS-MP		Ratio	EQ		PS-MP		Ratio
	Count	PNC	Count	PNC		Count	PNC	Count	PNC	
Mix 1	686	1.37 × 10^5^	197	3.93 × 10^4^	3.48	871	1.21 × 10^5^	244	3.38 × 10^4^	3.59
Mix 2	524	1.32 × 10^5^	433	1.09 × 10^5^	1.21	856	1.19 × 10^5^	867	1.20 × 10^5^	0.99
Mix 3	355	1.41 × 10^5^	471	1.87 × 10^5^	0.75	844	1.17 × 10^5^	1224	1.70 × 10^5^	0.69

Transport efficiency, or imaging efficiency for FIA, was also compared to evaluate the PNCs obtained. In FIA, efficiency is calculated using the fluid volume imaged, or the volume that is in the field of view of the camera, divided by the total volume processed. This efficiency relies on knowing the magnification of the objective (to determine volume in the FOV of the camera), the flow rate, and the total time of analysis. The number of images of particles taken by the camera can be divided by the volume imaged to determine PNCs. FIA efficiencies of the three mixtures ranged from 11 to 12%. In sp-ICP-TOFMS, the TE was calculated to be slightly higher, at 14.4%. This may explain the slight differences in the number of particles detected between the two techniques. However, even with different volumes used for analysis, the EQ beads remain a good way to determine stability and accurate particle number counting in each run, as the PNC should remain the same in all six analyses. The average PNC obtained across all three mixtures by FIA analysis was (1.36 ± 0.05) × 10^5^ and the mixture average PNC by sp-ICP-TOFMS was (1.19 ± 0.02) × 10^5^, which are in good overall agreement. The average relative difference obtained between the PNCs determined via FIA versus sp-ICP-TOFMS (for all particle types in each mixture) was 13.8%.

Taken together, these considerations highlight that despite fundamentally different detection mechanisms, reliable PNC determination in both FIA and sp-ICP-TOFMS requires careful control of particle flux to ensure reliable number-based quantification. The complementary use of both approaches therefore enables direct comparison of PNC results and provides valuable insight into potential sources of counting bias in complex particle systems.

### Complementary of FIA and sp-ICP-TOFMS

Having established analytical agreement between FIA and sp-ICP-TOFMS for spherical particle standards, Table 3 summarizes the complementary analytical outputs of both techniques and provides a framework for interpreting results for irregular, environmentally relevant particles, which will be presented in the next section. In this context, independent morphological information obtained by FIA provides direct comparison for these assumptions, enabling more confident interpretation of sp-ICP-TOFMS-derived particle size for spherical particles and nonspherical particles.

**Table 3:** Comparison of Analytical Outputs Obtained by FIA and sp-ICP-TOFMS

Analytical parameter	FIA	Sp-ICP-TOFMS
Particle Count	Number of particles directly imaged during a measurement run	Number of transient signals detected above a defined threshold in a measurement run
Transport efficiency (TE)	Depends on flow rate, imaging frequency, and particles properties (adhesion, aggregation...)	Dependent on sample introduction system, sample flow rate, and particle properties (adhesion, aggregation...)
Particle Number Concentration (PNC) [particles/mL]	Calculated by normalizing the number of imaged particles to the analyzed sample volume and dilution factor	Calculated by normalizing the number of detected particle events to the analyzed sample volume and corrected with TE and dilution factor
Particle shape	Directly measured from images; quantified using morphological descriptors (aspect ratio, circularity, elongation...)	Not accessible
Particle mass	Not accessible	Derived from signal intensity using appropriate quantification^60^
Particle size	Directly measured from images (equivalent circular diameter (ECD)...)	Calculated from particle mass assuming spherical shape and bulk density
Particle composition	Not accessible	Elemental and isotopic composition determined for detectable inorganic and metal constituents
Sample throughput	2 × 10^5^ particles mL^–1^	10^5^–10^6^ particles mL^–1^
Primary source of uncertainty	Focus	Transport efficiency, insufficient detectable signal, high ionic background

### Analysis of Environmentally Relevant Microplastics

Cryomilled microplastics from ocean-weathered plastics were analyzed using both FIA and sp-ICP-TOFMS. In contrast to well-defined commercial standards, cryomilled particles exhibit irregular shapes, broad size distributions, and heterogeneous morphologies, reflecting the fragmentation and weathering processes encountered in real-world environments. This sample was chosen for its complexity with regard to analysis by both FIA and sp-ICP-TOFMS. It is used here as a case-study to analyze real-world micro and nanoplastics present in oceanic environments, and whose degradation can have serious toxicologic implications.

The strength of sp-ICP-TOFMS lies in its simultaneous multielement detection capability. The particles detected from this plastic sample revealed multiple elemental transients with various intensities, reflecting a heterogeneous particle population (see Figure 6). Previous bulk characterization of the ocean-weathered plastic by acid digestion, followed by ICP-MS revealed the presence of metal additives such as Cd and Al, which are commonly used in plastics as pigments, stabilizers, and fillers.^55^ These additives can be investigated at the particle level, as shown in Figure 6. Indeed, the most common particles detected were Cd-only particles (*n* = 621), demonstrating that metal additives can serve as effective tracers for smaller plastic fragments that would otherwise remain invisible when monitoring carbon alone. Among the detected C-containing particles (*n* = 237), 19% were codetected with Cd, while smaller fractions (<2%) additionally contained Al (C–Cd–Al) or Cu (C–Cd–Al–Cu). In fact, the original plastic material consisted of a faded orange buoy, which historically have used Cd-based pigments (cadmium selenide sulfide) to produce orange coloration.

**Figure 6:**
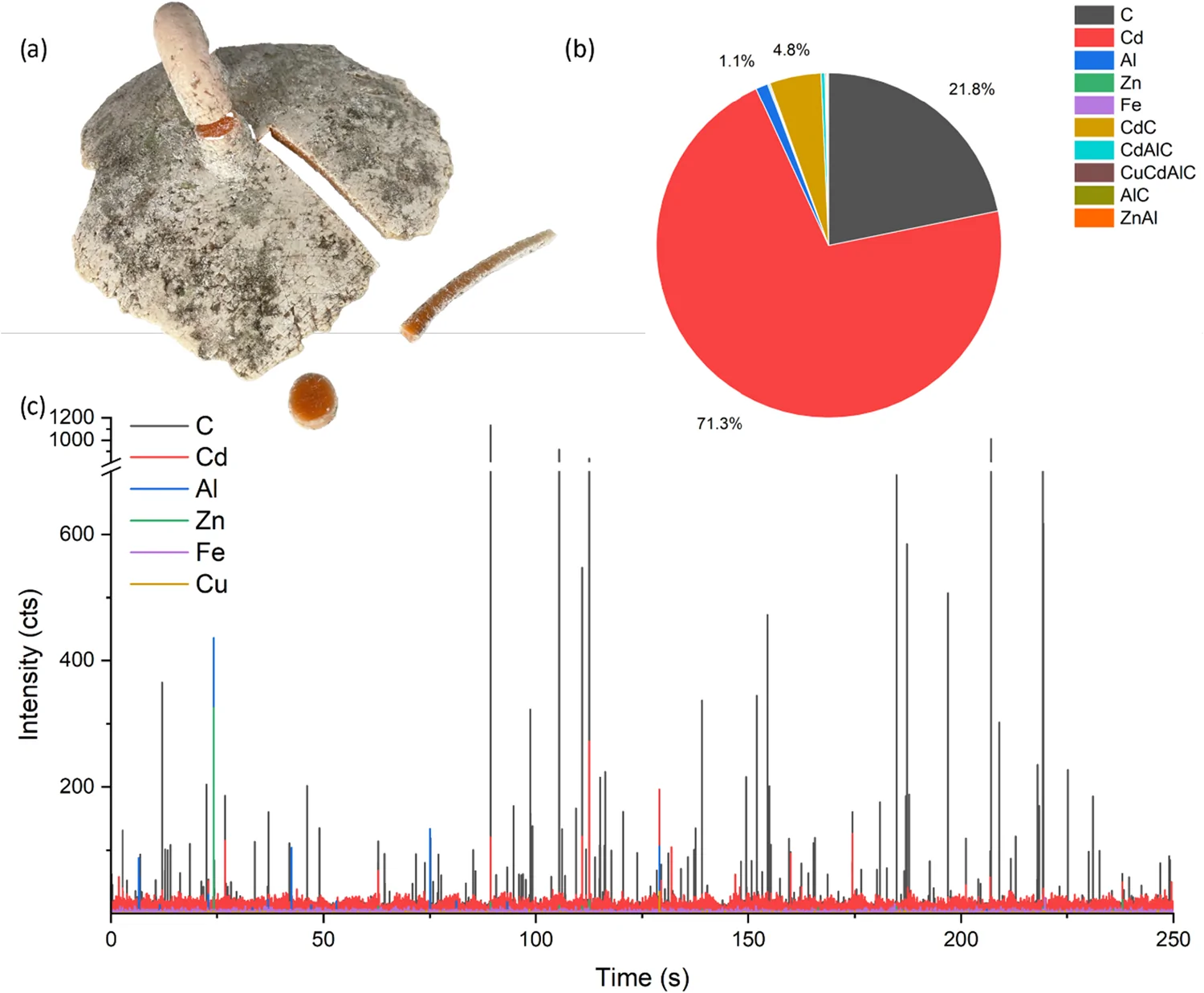
A weathered plastic buoy, shown in (a), was first cut into smaller pieces shown in the figure as the two fragments. Particles were generated from these fragments by cryomilling. Using sp-ICP-TOFMS, other metal additives are detected, such as Cd, shown in total by (b) via a pie chart as percentage of particles. This not only gives information on the plastic sample but also allows for other elements to be used to detect plastic particles that may fall below the detection limits of carbon on ICP-MS. Part of the time trace obtained for this sample is shown in (c).

While sp-ICP-TOFMS provides detailed compositional information at the single-particle level, particle size determination relies on conversion of measured elemental mass to an equivalent spherical diameter. To evaluate the validity of this assumption for ocean-weathered plastics, FIA measurements were performed to assess particle sizes and circularity determinations. Diameters obtained from FIA are displayed in Figure 7a, along with circularity metrics, and confirm that these particles deviate substantially from spherical geometry. FIA reveals a broad and heterogeneous size distribution (mean diameter 2 ± 1 μm; RSD ≈ 50%), consistent with the irregular particle shapes observed in the images (Figure 7a). Circularity values of the weathered plastic particles obtained by FIA are indicative of angular fragments and irregular shapes and range from 0.2 up to 1.0. Circularity assessment revealed that 29% of the detected particles can be considered spherical, 58% are angular fragments and 14% are elongated fragments, respectively. To showcase the capability of circularity as a discriminating feature, spherical EQ beads were added to the cryomilled plastic sample, and the mixture was analyzed by FIA. Figure 7b shows circularity versus diameter plot and image collage for the weathered plastic and EQ bead mixture. Despite this overlap in sizes obtained from plastics and the EQ beads, the spherical EQ beads form a clearly defined cluster at the expected diameter and circularity values (red arrow), consistent with that shown in Figure 3. Morphological differences are also evident in the FIA images collage, where the spherical EQ beads are easily distinguishable from the oblong, noncircular, and uneven surfaces of the plastic fragments,

**Figure 7:**
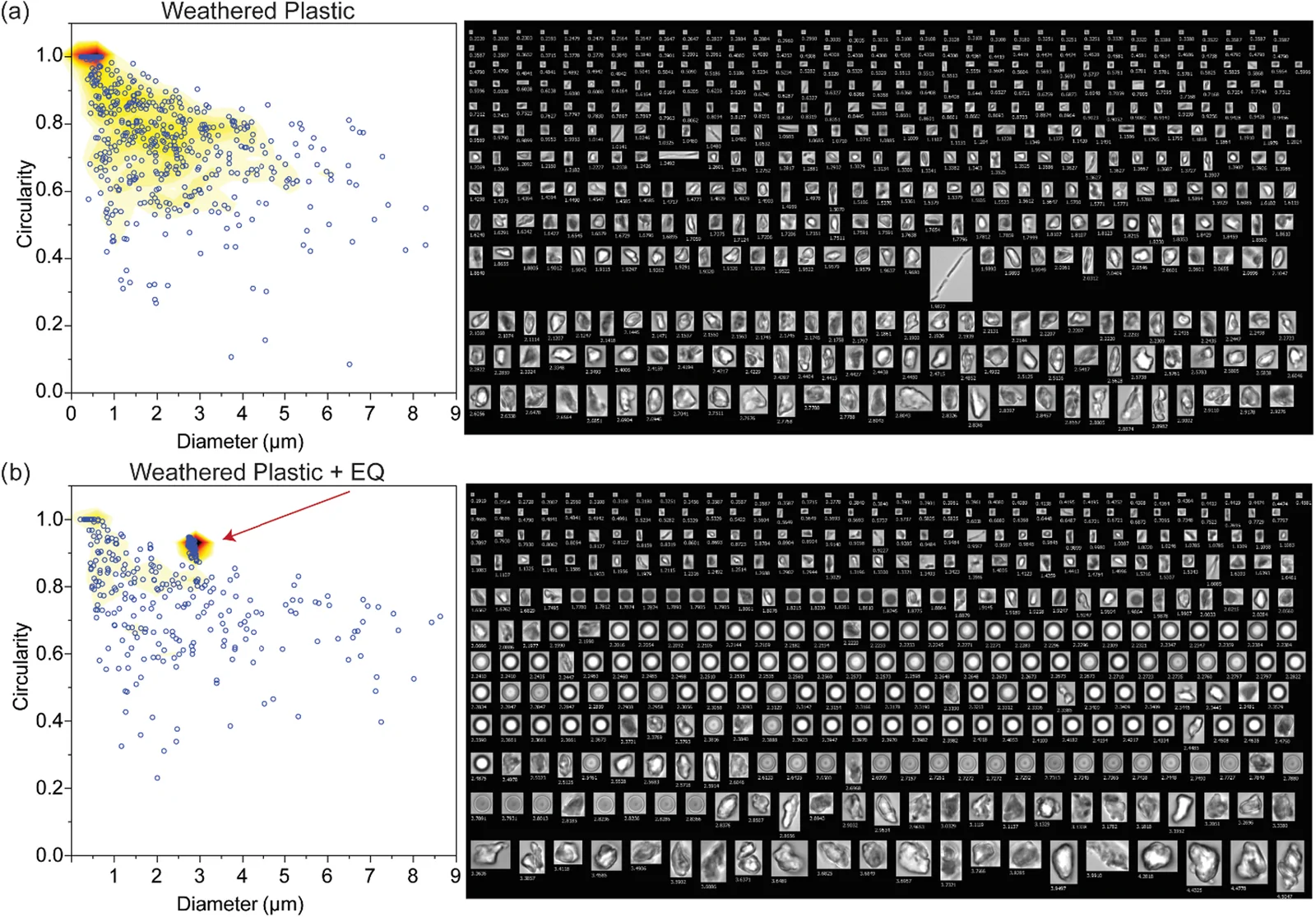
Circularities of the particles detected in the weathered sample (a) show large spread of particles across all diameters plotted. After the EQ standard is added (b) a clear population of spherical particles can be seen (shown by the red arrow), even with the large spread from weathered plastics. Blue dots overlaying the density plots are the actual data points. The irregular morphologies of these nano and micro plastics are highlighted in selected particle images from FIA.

These results demonstrate that the spherical geometry assumption typically used for mass-to-size conversion in sp-ICP-TOFMS is not valid for environmentally relevant MPs. Under these conditions, particle mass distributions from sp-ICP-TOFMS data represent a more appropriate and robust metric. If a size conversion is warranted, mass-derived particle sizes should be interpreted strictly as equivalent spherical diameters rather than true physical dimensions. For example, a simple cubic particle would produce a spherical equivalent diameter approximately 24% larger than its true edge length, while irregular environmental fragments are likely to produce even larger and less predictable deviations. Importantly, two particles with identical mass but distinct morphologies, e.g., a compact fragment and an elongated fiber, would yield the same mass-derived spherical size. For these particles, the underlying spherical assumption applied in sp-ICP-TOFMS is invalid, highlighting the importance of independent morphological information. To show how dependent accurate sp-ICP-TOFMS results are on using a known particle geometry, Figure 8 shows the estimated diameters of these plastic particles obtained via FIA, as well as from sp-ICP-TOFMS with an assumed-spherical shape using the mass of carbon.

**Figure 8:**
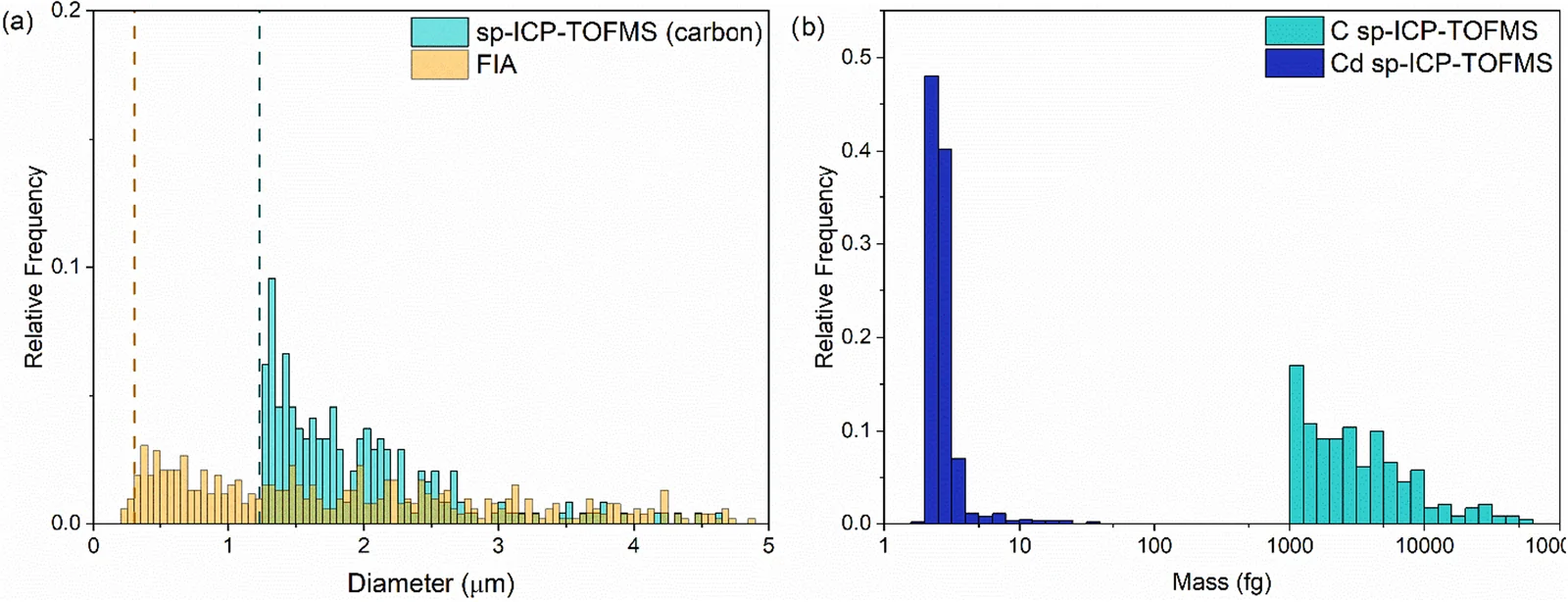
Comparison of the particle size distributions obtained by FIA (orange) and mass-derived assumed-spherical sizing from sp-ICP-TOFMS (blue) using carbon are shown in (a). FIA sizes represent all particles optically detected, independent of composition while sp-ICP-TOFMS detection is composition-specific. Dashed lines represent the LODs for both methods. For irregular particles, reporting particle mass may be a more appropriate metric than converting to an equivalent spherical diameter. The mass histograms of C and Cd detected from all particle events detected with either element is shown in (b), where mass of C is in light blue, and mass of Cd is in dark blue.

The population of particles analyzed by each technique must also be considered to directly compare size distributions obtained with carbon alone. Besides revealing nonspherical morphologies, FIA also shows a substantial number of particles exhibit diameters below ∼1 μm, which is well below the detection limit for carbon by sp-ICP-TOFMS (∼1.5 μm), as seen by the truncated size distribution in Figure 8. While accurate sizing of this sample may be infeasible using the standard spherical-assumption method with carbon by sp-ICP-TOFMS, there is still valuable information in the quantification of mass of carbon per particle, and detection of other elements of interest. To illustrate this, the mass of Cd detected in particles is plotted in Figure 8b alongside the mass of carbon in all particles detected with C. Cd was the most prominent particle-type detected, and its histogram follows a long-normal trend.

Figure 8 illustrates that the techniques sampled different particle populations, and direct comparison of size distributions does not represent a one-to-one correspondence. Considering the detection limits for Cd (2.3 fg) and C (1.1 pg), together with the observed distribution shape and truncation of the carbon signal (Figure 8a), the Cd-only detections likely correspond to nanoplastics particles (<1 μm) that were not captured in the carbon-based sp-ICP-TOFMS analysis. The carbon signal falls below the carbon-specific LOD in sp-ICP-TOFMS (as indicated by the dashed diameter threshold in Figure 8a). Consequently, no particle size estimation combining Cd and C was attempted, given the lack of prior knowledge on pigment composition and plastic formulation. Nevertheless, this smaller particle fraction helps now to explain the “missing” fraction when comparing FIA and sp-ICP-TOFMS.

Together, these results highlight the complementarity of FIA and sp-ICP-TOFMS for the characterization of environmentally weathered microplastic particles. While sp-ICP-TOFMS enables the detection of chemically distinct and potentially hazardous particles through multielement analysis, FIA provides the morphological context necessary to interpret particle size and shape distributions. In particular, FIA-derived shape descriptors such as circularity allow direct assessment of the validity of spherical mass-to-size assumptions commonly applied in sp-ICP-TOFMS analyses. Whereas spherical standards form well-defined clusters consistent with this assumption, irregular environmental particles exhibit substantial deviations from spherical geometry. Combining both techniques provides a more complete and reliable characterization of environmentally relevant microplastic particles.

## Conclusions

In contrast to previous multimethod studies primarily focused on particle number concentration of spherical microplastic standards, the present work emphasizes the role of particle morphology in validating sp-ICP-TOFMS-based size and mass quantification, with particular attention to irregular and nanoscale plastic particles. It is important to consider that real-world samples will not be perfectly spherical and have degradation that cannot be accounted for with just sp-ICP-TOFMS alone. Using FIA and sp-ICP-TOFMS together allows for confirmation, visually, of particle shape and morphology while also being quantitative for carbon and other elements within particles.

Using these two methods in parallel as complementary techniques could improve the understanding of particle populations within complex matrices; particle populations that were previously not identified were uncovered via FIA by not relying on a spherical assumption, as shown by plastic particles. While this manuscript focused on nano- and microplastic applications, the approach can be used for other particle types, given they are within the size range and detection limits for each method. This approach could also be used for single cell studies and could allow for the distinction between intact and burst cells. The results demonstrate that the sequential combination of flow imaging and sp-ICP-TOFMS provides consistent and complementary information for single particle characterization. Overall, while showcased here using sp-ICP-TOFMS, the inclusion of morphological information strengthens single-particle ICP-MS analysis, independent of the mass analyzer used.

This work emphasizes the importance of combining these techniques, as information from both methods is needed to obtain a complete and comprehensive analysis of these plastic samples. Future work will focus on direct coupling of both techniques for real-time multimodal analysis to enable verification of morphology-dependent differences in particle composition and improve the accuracy of particle mass and size estimations.

## Supplementary Material



## Notes

Additional FIA collage images, mass spectral time traces, and sizing histograms obtained for the particle suspensions (PDF)
