# Supplementary material for: Integrating Flow Imaging Analysis and Single-Particle ICP-TOFMS for Comprehensive Micro- and Nanoplastic Characterization
Source: Anal Chem. 2026 Aug 13;98(33):24200–14. doi: 10.1021/acs.analchem.6c02328 (PMC13523551; doi:10.1021/acs.analchem.6c02328)
Supplement: Supplementary file 1 [file ac6c02328_si_001.pdf]

Supporting Information for

Integrating Flow Imaging Analysis and Single-Particle ICP-TOFMS for comprehensive micro- and nano-plastic characterization

Sarah E. Szakas[1¥*], Lyndsey Hendriks[2¥*], Gunda Koellensperger, Benjamin T. Manard[1*]

1. Chemical Sciences Division, Oak Ridge National Laboratory, Oak Ridge, TN, 37830, USA
2. University of Vienna, Faculty of Chemistry, Institute of Analytical Chemistry, Waehringerstrasse 38, 1090 Vienna, Austria

¥Both Authors contributed equally and should be considered first authors

*Corresponding Authors: B.T. Manard, manardbt@ornl.gov; S.E. Szakas, szakasse@ornl.gov; L. Hendriks, lyndsey.hendriks@univie.ac.at

Table of Contents for Supporting Information

Table S1 Summary of the particle standards used for method validation 1
Table S2 Operating conditions for sp-ICP-TOFMS 2
Figure S1 Collage of images obtained via FIA for Eu-NPs. 3
Figure S2 Collage of images obtained via FIA for Fe-MPs. 3
Figure S3 Collage of images obtained via FIA for EQs. 4
Figure S4 Collage of images obtained via FIA for PS-MPs. 4
Figure S5 Example time traces of individual particle runs. 5
Figure S6 Histogram of the 100 nm Au particles 6
Figure S7 Time trace of the Mixture 1 7
Table S3 Sensitivities 7

Table S1 Summary of the particle standards used for method validation

	Eu-NP	Fe-MP	EQ	PS-MP

Diameter* [µm]	0.3± 0.03	1 ± 0.05	3.1+/- 0.1	5.07± 0.09
Particle number concentration* (particles per mL)	6.812 x 10^{11}	7-10 x 10^{9}	3.3 x 10^{5}	10% by weight
Plastic Type*	PS	-	PS	PS
Elemental composition	1.68 % Eu	Mixture of maghemite and magnetite 26% Fe	Traces of Ce, Eu, Ho and Lu	-

*Specified by manufacturer

Table S2 Operating conditions for sp-ICP-TOFMS

Instrument Parameter	Value
Sample introduction system	Cytoneb (ESI)
Injector [mm]	Quartz 1.5
Sample uptake [µL min^{-1}]	10
Nebulizer gas [L min^{-1}]	0.50
Additional gas [L min^{-1}]	0.50
Auxiliary [L min^{-1}]	0.74
Cool Gas [L min^{-1}]	14.3
Sampling Depth [mm]	6.0
RF Power [W]	1550
CCT Mass [V]	72.0
CCT Bias [V]	-2.2
Notch amplitude [V]	200
Notch filter [m/z (V)]	35(1.0), 17.5(2.0), 15(2.0), 40(0.8)

Figure S1 Collage of images obtained via FIA for Eu-NPs.

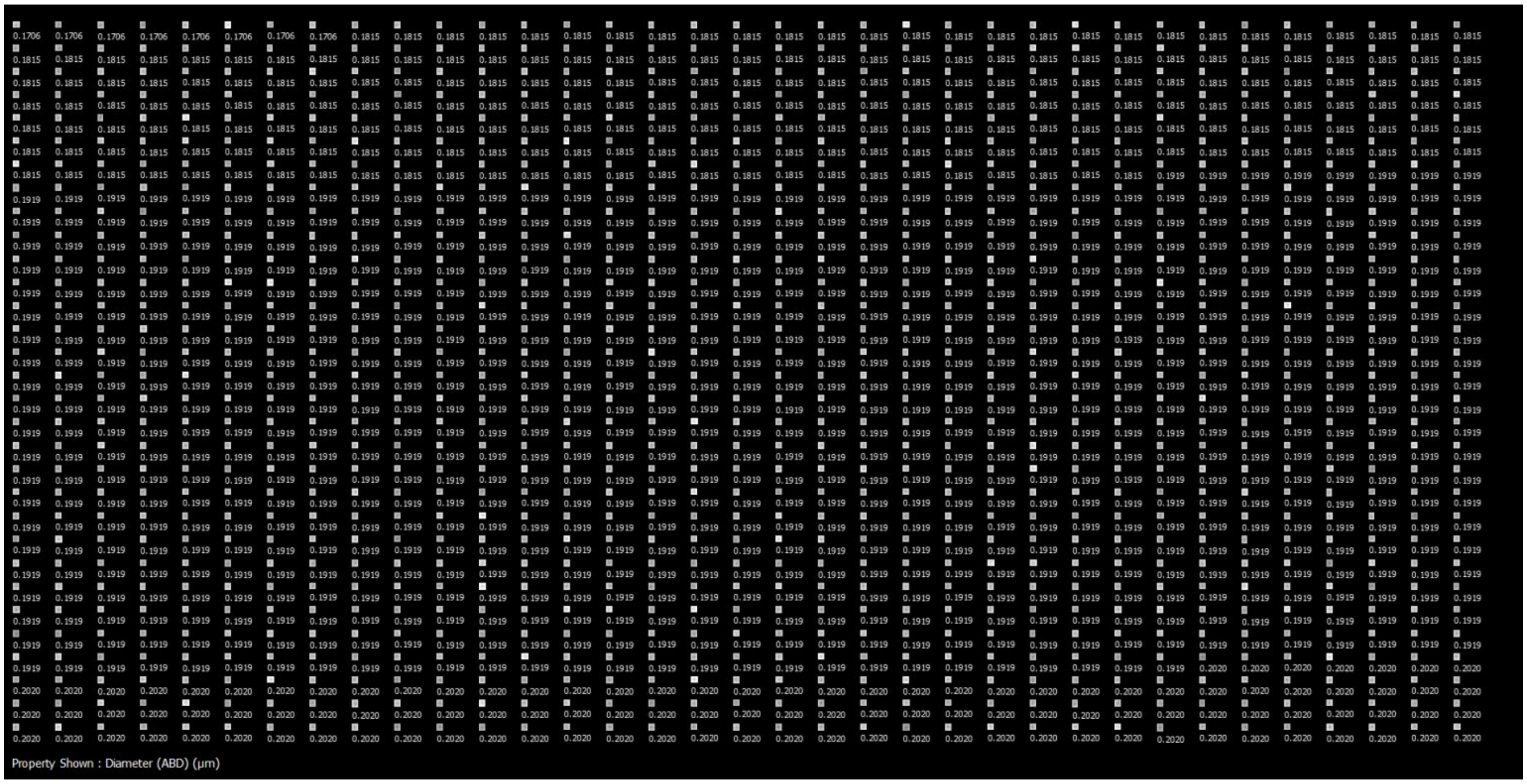

Figure S2 Collage of images obtained via FIA for Fe-MPs.

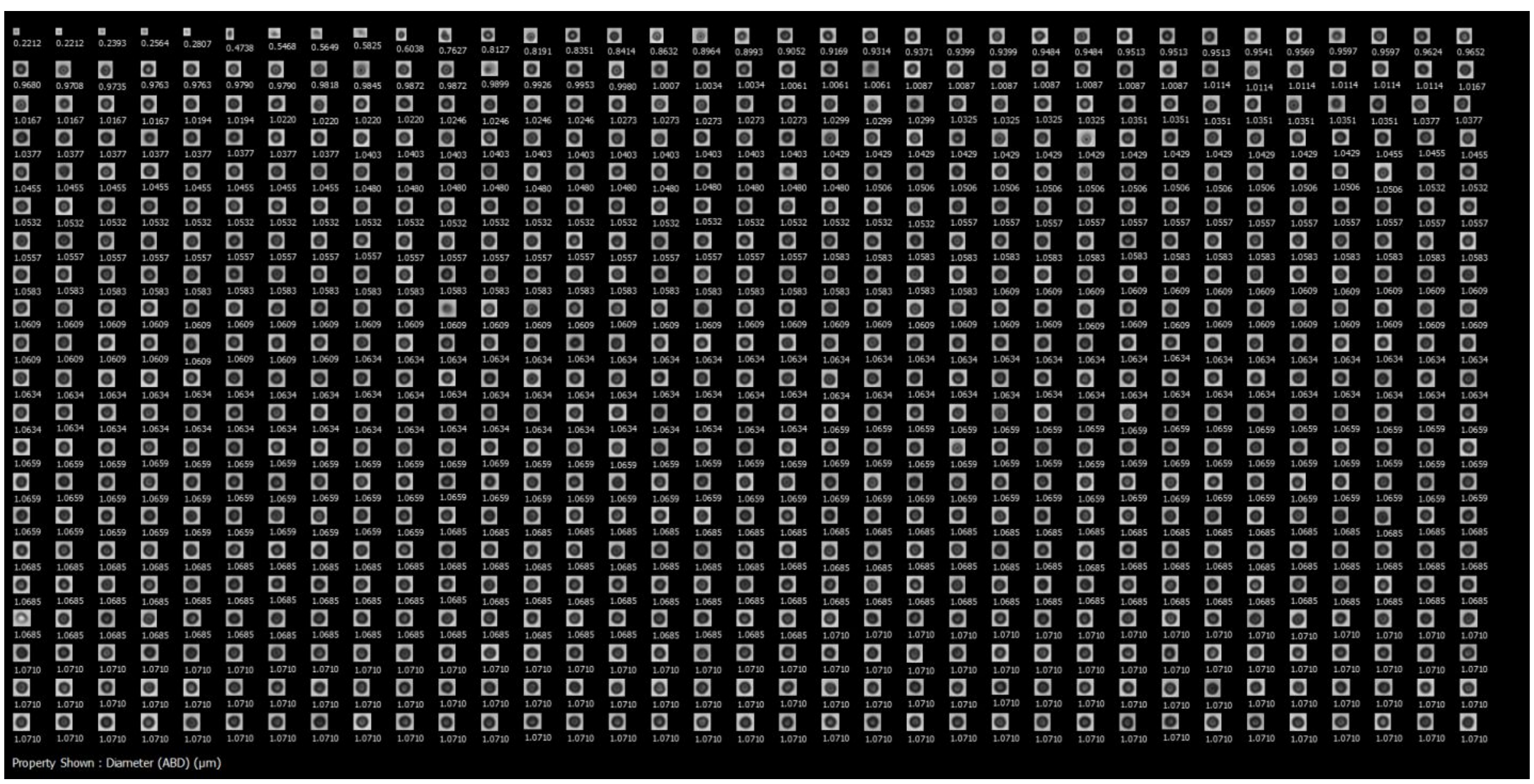

Figure S3 Collage of images obtained via FIA for EQs.

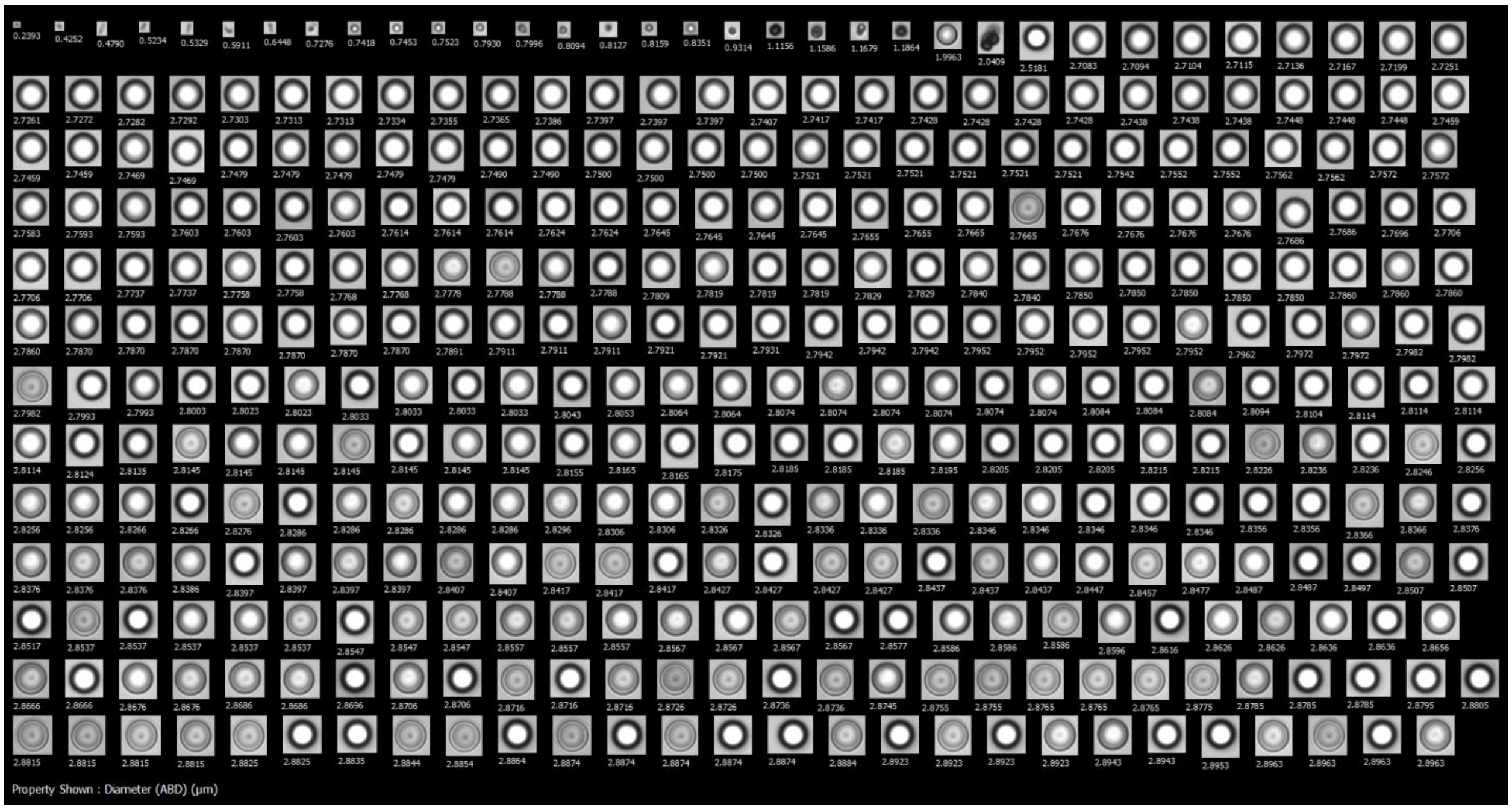

Figure S4 Collage of images obtained via FIA for PS-MPs.

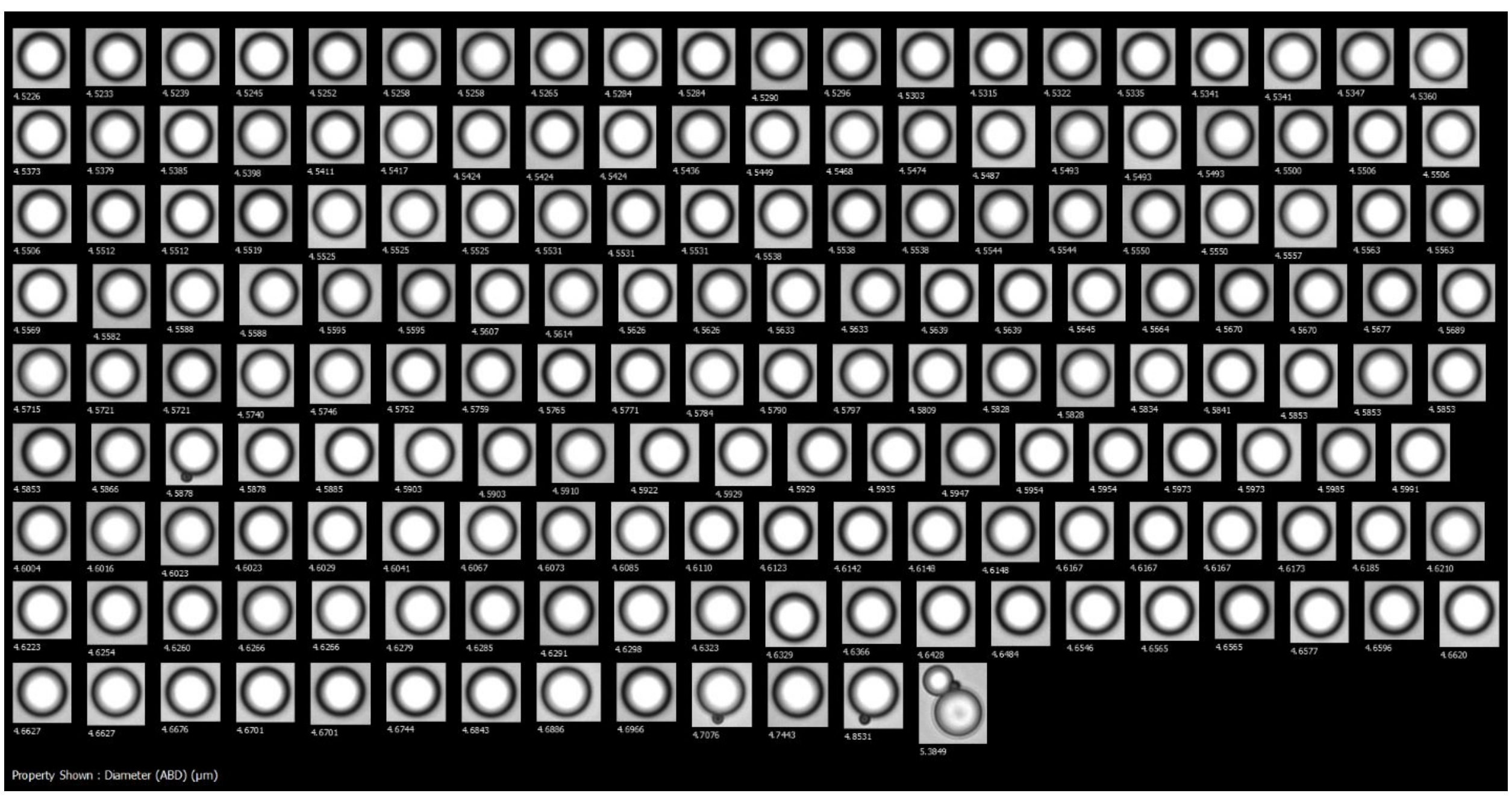

Figure S5 Example time traces (30s in total) of the individual single particle runs from each of the four beads used in the main text.

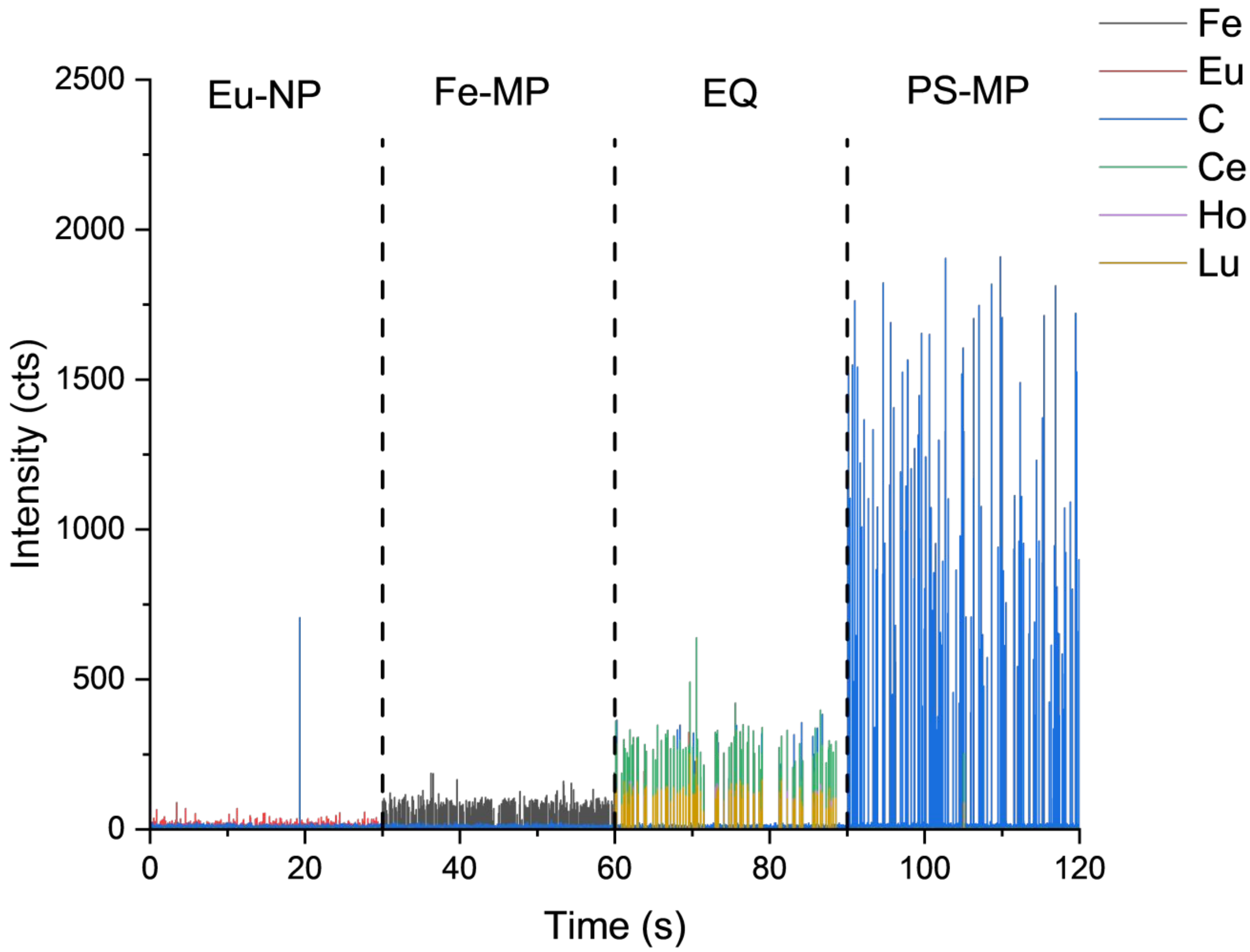

Figure S6 Histogram of the 100 nm Au particles used to calculate the transport efficiencies.

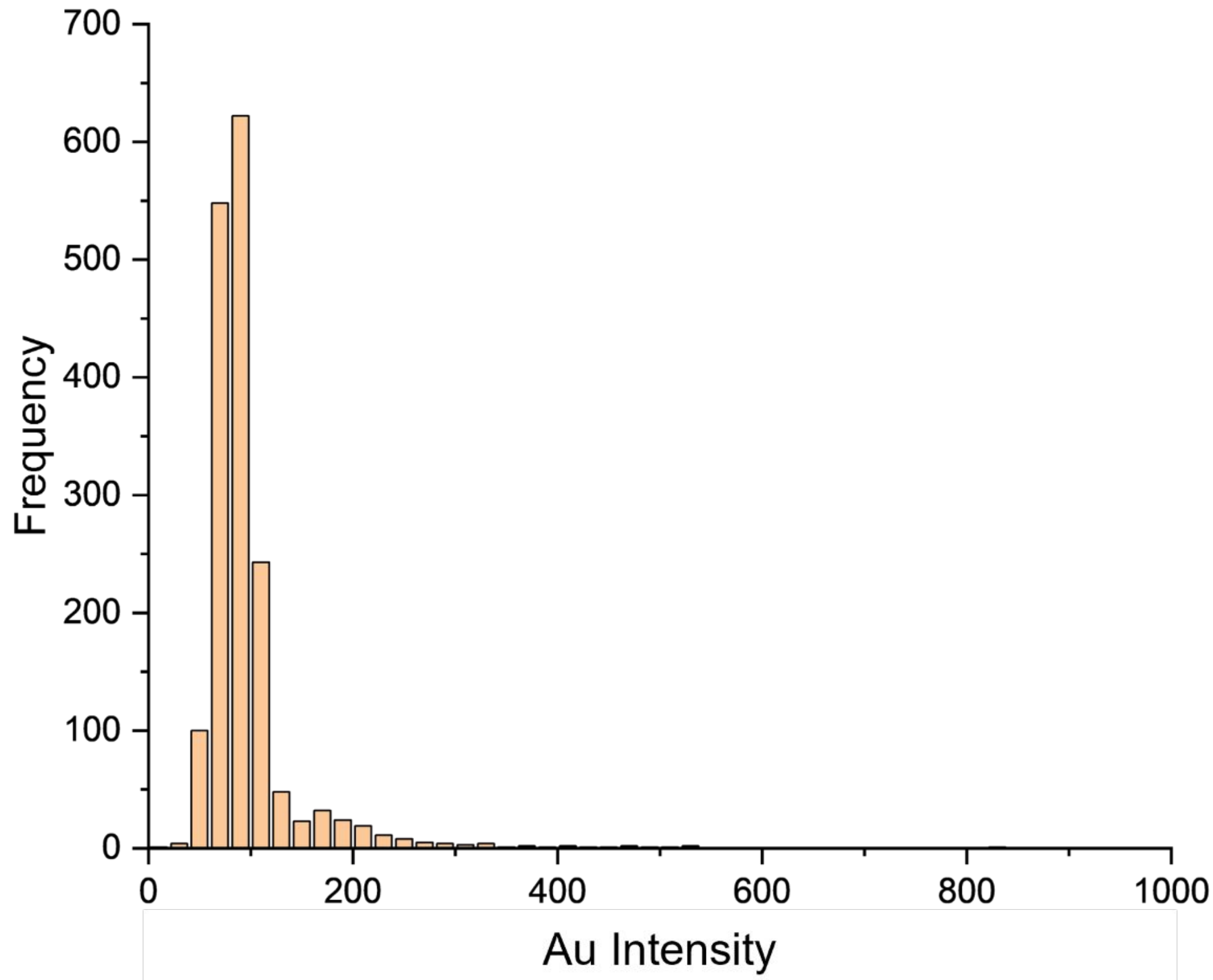

Figure S7 Time trace of the Mixture 1 (of the EQ and PS-MP beads) showing both high intensity carbon-only particles (blue) as well as lower intensity carbon particles concurrently detected with elements doped into the EQ beads (Eu, Ce, Ho, Lu).

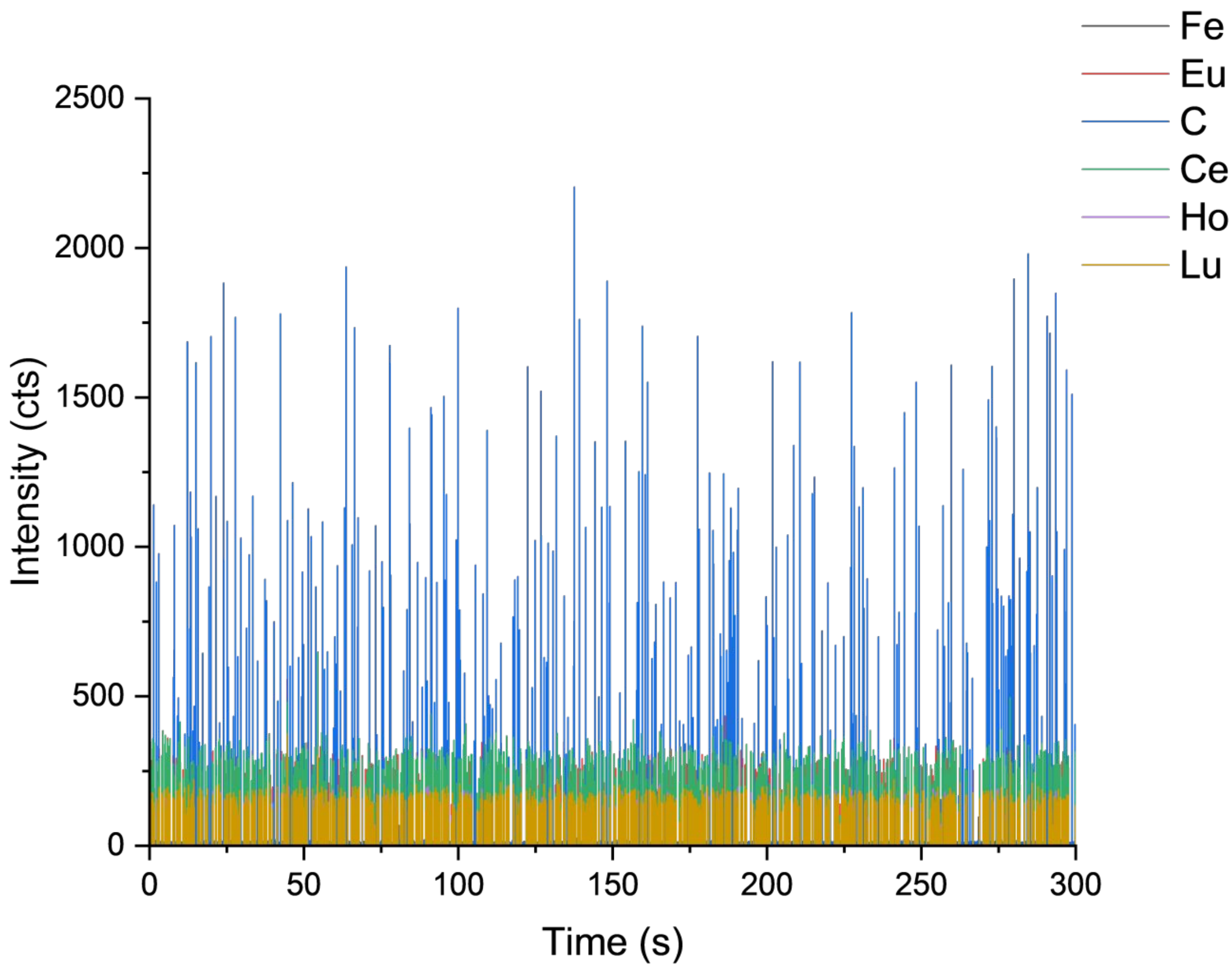

Table S3

Isotope of Interest	Sensitivity (cts / g)
^{12}C	2.26×10^{13}
^{57}Fe	5.41×10^{14}
^{151}Eu	7.06×10^{16}
